# Root Growth Adaptation is Mediated by PYLs ABA Receptor‐PP2A Protein Phosphatase Complex

**DOI:** 10.1002/advs.201901455

**Published:** 2019-12-11

**Authors:** Yang Li, Yaping Wang, Shutang Tan, Zhen Li, Zhi Yuan, Matouš Glanc, David Domjan, Kai Wang, Wei Xuan, Yan Guo, Zhizhong Gong, Jiří Friml, Jing Zhang

**Affiliations:** ^1^ State Key Laboratory of Plant Physiology and BiochemistryCollege of Biological Sciences China Agricultural University Beijing 100193 China; ^2^ Institute of Science and Technology Austria Am Campus 1 3400 Klosterneuburg Austria; ^3^ State Key Laboratory of Crop Genetics and Germplasm Enhancement and MOA Key Laboratory of Plant Nutrition and Fertilization in Lower‐Middle Reaches of the Yangtze River Nanjing Agricultural University Nanjing 210095 China

**Keywords:** *Arabidopsis*, PIN phosphorylation, PP2A, PYLs, root adaptation

## Abstract

Plant root architecture dynamically adapts to various environmental conditions, such as salt‐containing soil. The phytohormone abscisic acid (ABA) is involved among others also in these developmental adaptations, but the underlying molecular mechanism remains elusive. Here, a novel branch of the ABA signaling pathway in *Arabidopsis* involving PYR/PYL/RCAR (abbreviated as PYLs) receptor‐protein phosphatase 2A (PP2A) complex that acts in parallel to the canonical PYLs‐protein phosphatase 2C (PP2C) mechanism is identified. The PYLs‐PP2A signaling modulates root gravitropism and lateral root formation through regulating phytohormone auxin transport. In optimal conditions, PYLs ABA receptor interacts with the catalytic subunits of PP2A, increasing their phosphatase activity and thus counteracting PINOID (PID) kinase‐mediated phosphorylation of PIN‐FORMED (PIN) auxin transporters. By contrast, in salt and osmotic stress conditions, ABA binds to PYLs, inhibiting the PP2A activity, which leads to increased PIN phosphorylation and consequently modulated directional auxin transport leading to adapted root architecture. This work reveals an adaptive mechanism that may flexibly adjust plant root growth to withstand saline and osmotic stresses. It occurs via the cross‐talk between the stress hormone ABA and the versatile developmental regulator auxin.

## Graphical abstract

A novel branch of the ABA signaling pathway in *Arabidopsis* involving PYLs ABA receptor‐PP2A protein phosphatase complex, which modulates root gravitropism and lateral root formation through regulating phytohormone auxin transport, is identified. This work reveals an adaptive mechanism that may flexibly adjust plant root architecture to avoid the damage resulting from environmental stresses.

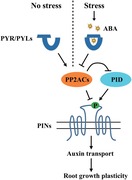

## 1 Introduction

Plants, unlike animals, cannot escape from environmental stresses and therefore have evolved endogenous mechanisms to adapt to detrimental conditions. Plant root development is tightly controlled by a range of external stimuli. For instance, salt and osmotic stresses induce the agravitropic root response and inhibit lateral root development.^1^, ^2^, ^3^, ^4^, ^5^ Reduced root gravitropism and branching might serve as an important adaptive mechanism through which plants growing in diverse natural conditions regulate root architecture to avoid the damage resulting from salt and osmotic stresses in the soil. Despite the importance of such adaptation, the underlying molecular mechanism remains to be investigated.

The plant hormone abscisic acid (ABA) accumulates rapidly under unfavorable conditions, such as hyperosmotic stress, and plays an important role in integrating a wide range of environmental cues and triggering a cascade of downstream stress responses. Binding of ABA to the PYRABACTIN RESISTANCE1 (PYR)/PYRABACTIN RESISTANCE1‐LIKE (PYL)/REGULATORY COMPONENT OF ABA RECEPTOR (RCAR) family of ABA receptors (abbreviated as PYLs) triggers a conformational change in PYLs that facilitates interactions with clade A protein phosphatase 2C (PP2C) members.^6^, ^7^ These interactions inhibit the activity of PP2Cs and thus relieve their inhibitory effects on downstream protein kinases, such as SUCROSE NON‐FERMENTING‐1 (SNF1)‐RELATED PROTEIN KINASEs (SnRKs), GUARD CELL HYDROGEN PEROXIDE‐RESISTANT1 (GHR1), CALCIUM‐DEPENDENT PROTEIN KINASEs (CDPKs), and CALCINEURIN B‐LIKE PROTEIN (CBL)‐INTERACTING PROTEIN KINASEs (CIPKs), allowing them to phosphorylate a range of downstream proteins that initiate ABA responses.^6^, ^7^, ^8^, ^9^, ^10^, ^11^, ^12^, ^13^, ^14^ To date, regulation of ABA signaling in many plant developmental processes is mainly dependent on this classical PYLs‐PP2C signaling module.

Previous studies have reported the functional roles of protein phosphatase 2A (PP2A) in ABA signaling.^15^, ^16^, ^17^, ^18^, ^19^, ^20^ Among these, mutation of PP2A scaffolding A subunit gene *ROOTS CURL IN NAPHTHYLPHTHALAMIC ACID1* (*RCN1*) results in a reduced ABA sensitivity in seed germination and stomatal closure,^17^ whereas the catalytic subunit mutant *pp2ac2* has ABA hypersensitivity in seed germination, root growth, and seedling development.^18^ Several PP2A subunits interact with ABA‐activated SnRK2‐type protein kinases.^20^ ABA prevents the formation of active PP2A holoenzyme.^16^ ABA‐mediated *Arbuscular mycorrhizal* colonization is also dependent on PP2A regulatory B subunit.^15^ Although much is known about the connection of PP2A and ABA signaling in plants, the molecular mechanism by which ABA controls PP2A activity is conceptually unclear.

In this study, we demonstrate that ABA restrains root gravitropism and lateral root formation under salt or osmotic stress via a novel branch of the ABA signaling pathway, which involves a complex of the PYLs ABA receptor and PP2A. In the absence of stress, PYLs promote PP2A activity, thus counteracting PINOID (PID)‐mediated phosphorylation of PIN‐FORMED (PIN) proteins, which facilitates polar auxin efflux from cells. Under stress, ABA binds to PYLs and PP2A activity is inhibited, thereby increasing phosphorylation of PIN proteins and in turn inhibiting directional auxin transport activity to contribute to ABA‐ and stress‐disturbed root architecture. This molecular mechanism allows plants to adjust their root developmental program to avoid damage under salt or osmotic stress conditions.

## 2 Results

### 2.1 PYLs‐Dependent ABA Signaling Modulates Auxin‐Mediated Root Architecture

A flexible, plastic root system allows plants to adapt to salt and osmotic stresses. Saline and osmotic conditions promote ABA production,^21^ and thus ABA may contribute to the adaptations of root growth to salt and osmotic stresses. It has been established that mutants defective in ABA biosynthesis develop more lateral roots and increased ABA inhibits lateral root development.^22^, ^23^ In agreement with these reports, ABA treatments led to a pronounced decrease in the density of both initiated primordia and emerged lateral roots in wild‐type *Arabidopsis thaliana* plants (Figure S1a, Supporting Information). A mutant lacking four ABA receptors (*PYR1, PYL1, PYL2*, and *PYL4*; abbreviated as *1124* mutant) was less sensitive to ABA than the wild type in terms of lateral root formation (Figure S1a, Supporting Information). A higher‐order mutant lacking five ABA receptors (*PYR1, PYL1, PYL4, PYL5*, and *PYL8*; abbreviated as *11458* mutant) was also completely resistant to ABA (Figure S1b,c, Supporting Information),^24^, ^25^ and showed increased lateral root density and impaired gravitropic root growth even under normal growth conditions (Figure S1b,c, Supporting Information). Moreover, around 3% (*n* = 403) of the *11458* mutant plants developed irregular cotyledons, characterized by one, three, or fused cotyledons (Figure S1d, Supporting Information). These defective phenotypes suggest that PYLs have redundant roles in plant growth and development. ABA INSENSITIVE1 (ABI1), which is one of protein phosphatases in clade A PP2C family, is predominantly expressed in the roots.^26^ The well‐established ABA‐insensitive dominant mutant *abi1‐1* (in the Col‐0 background)^27^, ^28^ is less sensitive to ABA in terms of lateral root elongation.^2^, ^29^, ^30^ However, we found that in response to different doses of ABA, this mutant showed normal sensitivity as wild type with respect to lateral root formation (Figure S1a,e, Supporting Information). Consistently, stably overexpressing *ABI1* in the transgenic plants (*ABI1‐OE*)^27^, ^31^ did not affect the sensitivity of lateral root to ABA in comparison with the wild type (Figure S1f, Supporting Information). Likewise, the ABA‐hypersensitive *abi1‐3* loss‐of‐function mutant^32^ showed no distinct lateral root phenotype compared with the wild type (Figure S1g, Supporting Information). Moreover, we observed that in contrast to the *1124* ABA‐receptor mutant, which was insensitive to salt and osmotic stresses during lateral root formation, the *abi1‐1* (Col‐0) mutant exhibited a normal lateral root reduction similar to the wild type (Figure S1h,i, Supporting Information).

**Figure 1:**
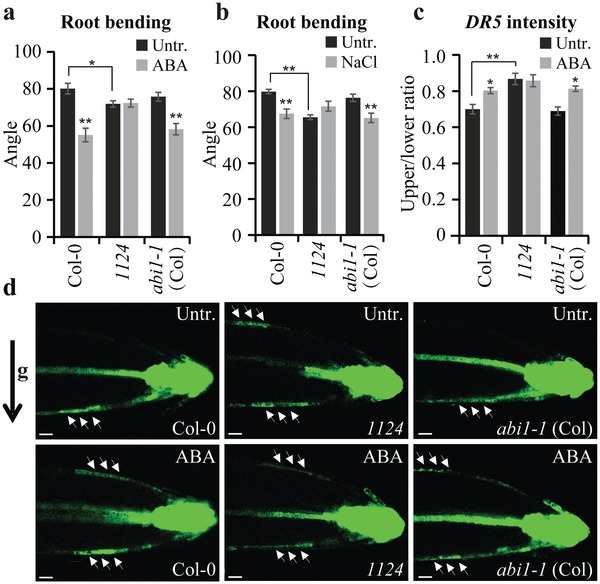
ABA affects root gravitropism and auxin relocation. a,b) Quantification of root gravitropic bending under ABA and NaCl treatments. Five‐day‐old seedlings were gravistimulated in the presence or absence of 30 × 10^−6^ m ABA (a) or 50 × 10^−3^ m NaCl (b) for 24 h (*n* ≥ 10 roots). The root growth angle after gravistimulation was measured as described.^34^ c,d) The effect of ABA on auxin translocation. Five‐day‐old seedlings expressing *DR5rev::GFP* were transferred to medium supplemented or not with 30 × 10^−6^ m ABA for 16 h and then gravistimulated for 4 h in darkness. Fluorescence resulting from *DR5rev::GFP* was used to monitor auxin translocation during the root gravitropic response (d). The ratio of mean fluorescence intensity of the upper to that of lower side of the root was quantified (c; *n* = 10 roots). The black arrow indicates the direction of gravity (*g*). The white arrows mark auxin flow. Scale bar: 60 µm. Error bars represent ± SE (a–c). (∗) *P* < 0.05, (∗∗) *P* < 0.01 (Student's *t*‐test). Three independent experiments were performed with similar results. Representative images are shown.

Next, we assessed the role of ABA in root gravitropic responses. ABA signaling has been proposed to attenuate root gravitropism in wild‐type plants,^33^ as indicated by decreased root gravitropic index (Figure S1j, Supporting Information) and root growth angle^34^ after gravistimulation (**Figure** **1**a; Figure S1k, Supporting Information). Accordingly, the *1124* mutant was insensitive to ABA in terms of root gravitropism (Figure 1a; Figure S1j,k, Supporting Information). Similar to its effects on lateral root formation, ABA inhibited gravitropic root growth in the ABA‐insensitive *abi1‐1* (Col‐0) mutant (Figure 1a; Figure S1j,k, Supporting Information) and *ABI1‐OE* transgenic plants (Figure S1l, Supporting Information). Furthermore, both the loss‐of‐function single mutant *abi1‐3* and the triple mutant *abi1 abi2 hab1* (abbreviated as *pp2c* mutant) defective in three PP2Cs,^35^ had a normal root gravitropism response to ABA (Figure S1m,n, Supporting Information). We further confirmed the involvement of ABA perception using transgenic plants expressing wild‐type *PYL1* or phospho‐mimic *PYL1S119D*, in which the ABA binding pocket is blocked, both driven by the native *PYL1* promoter.^36^ The ABA‐insensitive root gravitropic phenotype of *1124* was complemented by the wild‐type *PYL1* transgene, but not by *PYL1S119D* (Figure S1o, Supporting Information). Moreover, the *1124* mutant was insensitive to salt and osmotic stress‐induced inhibition of root gravitropism, whereas the *abi1‐1* (Col‐0) mutant exhibited normal sensitivity to these treatments (Figure 1b; Figure S1p,q, Supporting Information).

Lateral root formation, and particularly root gravitropism, are typical processes regulated by the asymmetric distribution of auxin.^37^, ^38^, ^39^ Therefore, we crossed the auxin‐responsive reporter *DR5rev::GFP* into *1124* and *abi1‐1* (Col‐0) mutant to indirectly monitor the gravity‐induced auxin redistribution after ABA treatment. As shown previously,^38^, ^40^ gravity stimulation induced asymmetric auxin distribution, with a strong *DR5* signal along the lower sides of the roots (Figure 1c,d). The ABA treatment markedly affected this gravistimulation‐induced auxin response asymmetry, resulting in *DR5* signal at both the lower and upper sides of the gravistimulated roots (Figure 1c,d). The *1124* mutant showed reduced *DR5* asymmetry even under untreated conditions, and did not respond to ABA treatment (Figure 1c,d), consistent with ABA insensitivity with respect to gravitropic bending (Figure 1a; Figure S1k, Supporting Information). By contrast, the *abi1‐1* (Col‐0) mutant showed similar ABA sensitivity in terms of *DR5* asymmetry to the wild type (Figure 1c,d), in line with the normal ABA sensitivity of this mutant in terms of gravitropic bending (Figure 1a; Figure S1k, Supporting Information). In agreement with the effect of ABA on auxin response, salt and osmotic stresses also disturbed the gravistimulation‐induced *DR5* asymmetry in the wild‐type and *abi1‐1* (Col‐0) mutant roots, but symmetric *DR5* signal in *1124* was not affected (Figure S1r,s, Supporting Information).

Taken together, these findings reveal a role for ABA perception by PYLs in the auxin‐dependent root adaptive development; nonetheless, this effect of ABA does not require PP2C phosphatase ABI1.

### 2.2 PYLs‐Dependent ABA Signaling Regulates PIN Distribution and Trafficking

**Figure 2:**
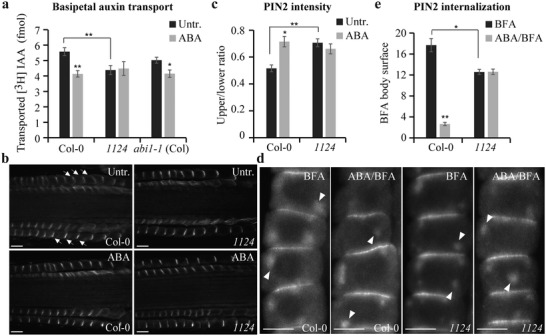
ABA affects PIN distribution and subcellular dynamics. a) The effect of ABA on root basipetal auxin transport. Six‐day‐old seedlings were subjected to 30 × 10^−6^ m ABA for 12 h. Basipetal auxin transport in the root was detected using [^3^H]‐labeled IAA (*n* = 12 roots). b,c) The effect of ABA on the gravistimulation‐mediated PIN2 gradient. PIN2 immunolocalization was performed in 4‐d‐old seedlings gravistimulated for 4 h in the presence or absence of 30 × 10^−6^ m ABA. Relative PIN2 signal intensity in the epidermis at the upper versus lower side of the root was quantified (c; *n* = 10 roots). Arrows mark PIN2 gradient. Scale bar: 10 µm. d,e) The effect of ABA on BFA‐induced PIN2 internalization. PIN2 immunolocalization was performed in 4‐d‐old seedlings pretreated or not with 30 × 10^−6^ m ABA for 3 h and then cotreated with 25 × 10^−6^ m BFA for 60 min. Mean surface area (pixels^2^) of BFA bodies per cell in root epidermis was quantified (e; *n* ≥ 62 cells). Arrowheads mark PIN2 internalized into BFA compartments. Scale bar: 10 µm. Error bars represent ± SE (a,c,e). (∗) *P* < 0.05, (∗∗) *P* < 0.01 (Student's *t*‐test). Three independent experiments were performed with similar results. Representative images are shown.

We next examined how ABA regulates the asymmetric distribution of auxin. The steady‐state *DR5* activity in the root tip was not influenced by ABA treatment (Figure S2a–c, Supporting Information), indicating that ABA does not affect auxin biosynthesis or the shoot‐to‐root delivery of auxin. The gravity‐induced asymmetric auxin distribution is mediated by shootward (basipetal) auxin transport.^40^, ^41^ Therefore, we tested whether ABA affects auxin flow using radioactively labeled indole‐3‐acetic acid ([^3^H]IAA). Indeed, in the ABA‐treated roots, the shootward [^3^H]IAA transport was inhibited compared with the untreated wild type (**Figure** **2**a). The *1124* mutant showed completely ABA‐insensitive shootward auxin transport compared with the wild type, but the *abi1‐1* (Col‐0) mutant retained normal sensitivity to ABA (Figure 2a). Consistent with the morphological phenotypes, this suggests that ABA regulation of auxin transport was mediated by PYLs but did not strictly require ABI1. Notably, the *1124* mutant showed reduced auxin transport without any treatment (Figure 2a), consistent with the impaired gravitropic response (Figure 1a; Figure S1k, Supporting Information) and gravity‐induced asymmetric auxin distribution of *1124* roots (Figure 1c,d) and with the defective growth of *11458* mutant seedlings (Figure S1b–d, Supporting Information).

The shootward auxin transport and gravity response are mediated by the activity of the PIN2 auxin transporter^40^, ^42^ and its dynamic, polar localization at the apical side of epidermal cells.^43^, ^44^ During the gravitropic response, PIN2 is differentially degraded in lytic vacuoles and the weaker PIN2 signal at the upper side versus the stronger signal at the root lower side presumably reinforces the asymmetric auxin flow required for the gravitropic response.^45^ Exposure to ABA prevented this gravity‐induced asymmetric PIN2 distribution (Figure 2b,c). The *1124* mutant was insensitive to this ABA effect, with no pronounced differential PIN2 asymmetry observed in gravistimulated roots that had been treated or not with ABA (Figure 2b,c). By contrast, the *abi1‐1* (Col‐0) mutant showed similar ABA sensitivity to the wild type (Figure S2d,e, Supporting Information). These observations are in agreement with the ABA inhibition of shootward auxin transport (Figure 2a), *DR5* asymmetry (Figure 1c,d), and root gravitropic bending (Figure 1a; Figure S1k, Supporting Information). Overall, PYLs receptors but not ABI1 phosphatase interfere with the gravity‐induced establishment of PIN2 asymmetry that is important for the gravitropic response.

The PIN proteins constitutively cycle between the plasma membrane and the endosomes, a process crucial for PIN polarity determination.^46^, ^47^ Recycling of PIN proteins from endosomes to the plasma membrane can be constrained specifically by brefeldin A (BFA), which leads to the internalization of dynamically cycling PIN proteins into BFA compartments.^48^ We next tested the effect of ABA on BFA‐sensitive PIN2 endocytic recycling. As documented previously,^48^ PIN2 proteins accumulated intracellularly after BFA treatment, but these BFA‐induced PIN2 internalizations were visibly attenuated by ABA treatment (Figure 2d,e; Figure S2f,g, Supporting Information). This inhibitory effect of ABA on BFA‐induced intracellular PIN2 accumulation was strongly abolished in the *1124* mutant (Figure 2d,e), but not in the *abi1‐1* (Col‐0) mutant (Figure S2f,g, Supporting Information). Notably, untreated *1124* roots already showed reduced PIN2 aggregation in BFA bodies (Figure 2d,e).

The PIN‐dependent auxin transport is also crucial for lateral root formation, where PIN1 is the major component.^49^ Because it is challenging to monitor and quantify the dynamic rearrangements of PIN1 polarity in lateral root primordia, we analyzed the effect of ABA on PIN1 distribution in the primary roots and emerged lateral roots. The predominant basal (rootward) PIN1 distribution in endodermis, pericycle, and stele cells^50^ was disrupted by external ABA supplementation. Generally, the basal polarity of PIN1 was less pronounced and resulted in an increased lateral distribution in both primary roots and emerged lateral roots (Figure S2h,i, Supporting Information). Again, as seen for other tested processes, the *1124* but not the *abi1‐1* (Col‐0) mutant was strongly insensitive to this ABA effect, and PIN1 more frequently localized to the lateral sides of cells in untreated *1124* roots as compared to that of the wild‐type control. (Figure S2h,i, Supporting Information).

Together, these results imply that ABA signaling modulates PIN polar distribution and trafficking via PYLs‐dependent and ABI1‐independent mechanism.

### 2.3 PYLs‐Dependent ABA Signaling Mediates PIN Phosphorylation

Next, we investigated how ABA signaling modulates PIN distribution and trafficking. Substantial pharmacological and genetic studies have shown that protein (de)phosphorylation is a crucial determinant for PIN polar targeting and recycling^51^, ^52^, ^53^, ^54^, ^55^, ^56^ and also for PIN activity.^57^, ^58^ We therefore assessed whether the phosphorylation status of PIN could be modified by ABA or altered in mutants defective in ABA perception.

**Figure 3:**
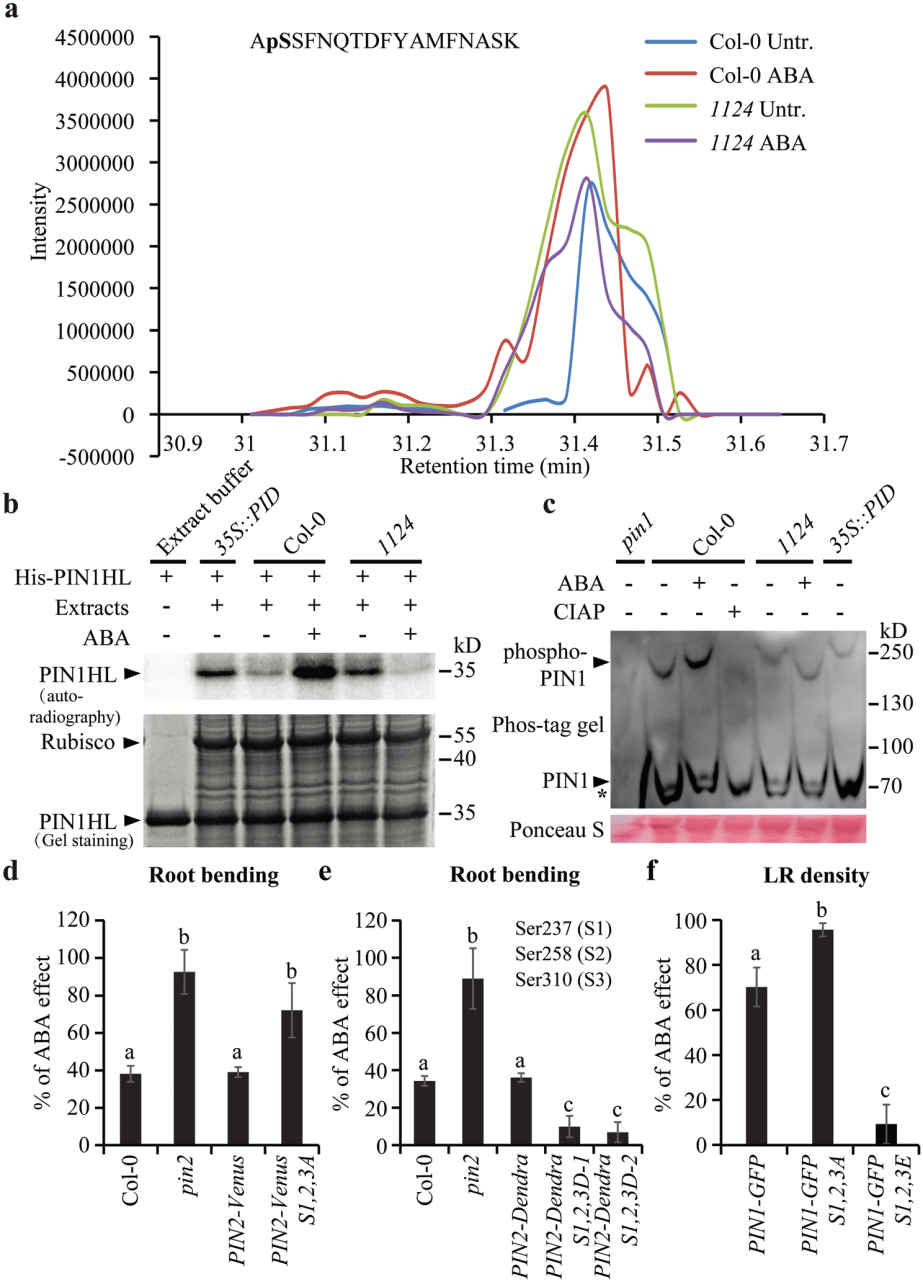
PYLs‐dependent ABA signaling regulates PIN phosphorylation status. a) Extracted ion chromatogram profile of phosphorylated peptide 257–273 derived from PIN2HL. Total protein extracted from 5‐d‐old seedlings treated or not with 30 × 10^−6^ m ABA for 4 h was coincubated with heterologously expressed GST‐PIN2HL, and then subjected to LC‐MS/MS on the tryptic peptides. b) An in vitro phosphorylation assay of PIN1HL. Equal amounts of total protein extracts from 5‐d‐old seedlings treated or not with 30 × 10^−6^ m ABA for 4 h were coincubated with heterologously expressed His‐PIN1HL, and then used for an in vitro phosphorylation assay. c) An in vivo phosphorylation profile after Phos‐tag mobility shift assay. Protein extracts from 7‐d‐old seedlings treated or not with 30 × 10^−6^ m ABA for 2 h were separated in a Phos‐tag gel. *pin1* mutant and *35S::PID* were used as negative and positive controls, respectively. Both phosphorylated and unphosphorylated PIN1 protein bands (arrowheads indicated) were detected with anti‐PIN1 antibody. * indicates nonspecific bands. CIAP was used as a positive dephosphorylation control. The experiments were repeated independently for three times, and similar results were always obtained, though the nonphosphorylated PIN1 bands were sometimes covered by the strong unspecific bands. Considering that all bands shifted abnormally for phos‐tag gels, the protein markers do not tell the real mobility for proteins. + and − indicate incubated with or without substrate, extracts, or treatment, respectively (b,c). d–f) Modification of ABA sensitivity by a phosphorylation‐based sequence. Five‐day‐old transgenic lines were gravistimulated with or without 30 × 10^−6^ m ABA for 24 h. The root growth angle was measured after gravistimulation (*n* ≥ 18 roots) and ABA effect on root bending was then evaluated (d,e). Two independent transgenic lines *PIN2‐Dendra S1,2,3D‐1* and *PIN2‐Dendra S1,2,3D‐2* were used (e). Seven‐day‐old transgenic lines germinated on MS medium with or without 0.3 × 10^−6^ m ABA were used for lateral root density quantification (*n* = 11 roots) and ABA effect on lateral root formation was then evaluated (f). Error bars represent ± SE. Means with different letters (d–f) are significantly different at *P* < 0.05 (Fisher LSD test). Three independent experiments were performed with similar results. Representative images are shown.

We extracted total proteins from seedlings, coincubated the proteins with the GST‐tagged PIN2 hydrophilic loop (GST‐PIN2HL) heterologously expressed in *Escherichia coli*, and performed liquid chromatography tandem‐mass spectrometry (LC‐MS/MS) on the tryptic peptides. One of the highest‐scored phosphorylated peptides we identified (Figure S3a, Supporting Information) showed a strong increase in abundance after ABA treatment (**Figure** **3**a; Figure S3b, Supporting Information). In the *1124* mutant, this phosphorylated peptide was already abundant and ABA treatment did not further enhance, but rather attenuated this phosphorylation (Figure 3a; Figure S3b, Supporting Information). Based on LC‐MS/MS analysis, the phosphorylation occurred at Ser 258 (Figure 3a; Figure S3a, Supporting Information), a site previously identified as relevant for PIN polarity and trafficking, and dependent on the PID protein kinase.^51^, ^53^ Thus, the LC‐MS/MS‐based analysis identifies a plausible phosphorylation site targeted by PYLs‐dependent ABA signaling.

Next, we used in vitro phosphorylation to further analyze the ABA‐mediated PIN phosphorylation. Equal amounts of the total protein extracted from wild‐type, *35S::PID*, and *1124* mutant plants were coincubated with the heterologously expressed His‐tagged wild‐type PIN1 hydrophilic loop (His‐PIN1HL), respectively, in an in vitro phosphorylation assay. After electrophoretic protein separation, a high amount of phosphorylated His‐PIN1HL was detected following incubation with the positive control (i.e., *35S::PID* protein extracts) (Figure 3b).^55^ Likewise, the total proteins extracted from the *1124* mutant had a markedly greater ability to phosphorylate His‐PIN1HL than those extracted from the wild type (Figure 3b). Notably, ABA treatment dramatically enhanced phosphorylation of His‐PIN1HL in the wild type; however, this effect was reduced when proteins extracted from the *1124* mutant were used (Figure 3b). These data indicate that PYLs‐dependent ABA signaling regulates PIN phosphorylation.

To further examine ABA regulation of PIN phosphorylation in vivo, we performed Phos‐tag mobility shift assay which could clearly separate phosphorylated proteins from the nonphosphorylated counterparts based on the migration speed of the corresponding bands.^59^ Proteins extracted from untreated and ABA‐treated wild‐type or *1124* seedlings were separated in a Phos‐tag gel and detected by immunoblot analysis using an anti‐PIN1 antibody. Under ABA treatment, the phosphorylated PIN1 band in wild‐type sample was shifted to a higher molecular weight (Figure 3c; Figure S3c, Supporting Information), which was abolished by the addition of calf intestinal alkaline phosphatase (CIAP) (Figure 3c). In comparison with the wild type, the phosphorylated PIN1 band in *1124* migrated slower and the presence of ABA rather accelerated its migration reflected by the clearly shifted bands (Figure 3c). As a positive control, an upshift of phosphorylated PIN1 protein was clearly detected in *35S::PID* (Figure 3c). These results fully support the data of LC‐MS/MS‐based analysis and in vitro phosphorylation assay. The opposite effects of ABA on PIN phosphorylation in the wild type and *1124* mutant imply that unknown feed‐back regulation of ABA signaling might be involved. However, these combined results suggest that ABA positively regulates PIN phosphorylation via PYLs.

To investigate the relevance of Ser 258 to the effect of ABA on root architecture, we used transgenic lines expressing phosphorylation‐deficient or phosphorylation‐mimic PIN2, in which Ser 258 and two surrounding Ser residues were mutated to Ala (*PIN2‐Venus S1,2,3A*) or Asp (*PIN2‐Dendra S1,2,3D*), respectively. Neither the phospho‐deficient nor phospho‐mimic constructs fully rescued the agravitropic phenotype of the *pin2* loss‐of‐function mutant.^51^ Therefore, we evaluated the ABA sensitivity of the gravitropic bending in these lines by transferring the 5‐d‐old seedlings to medium supplemented with or without ABA, straightening their roots, and subsequently subjecting them to gravistimulation for 24 h. Similar to the wild type, ABA treatment reduced gravitropic bending in the *PIN2‐Venus* roots (Figure 3d), but the phospho‐deficient *PIN2‐Venus S1,2,3A* lines showed even increased sensitivity to ABA (Figure 3d). By contrast, *PIN2‐Dendra* showed normal response to ABA, and the *PIN2‐Dendra S1,2,3D* phospho‐mimic lines were more insensitive to ABA in terms of gravitropic bending (Figure 3e). Accordingly, salt‐ and mannitol‐mediated inhibition of root gravitropism was again enhanced in *PIN2‐Venus S1,2,3A* but largely attenuated in *PIN2‐Dendra S1,2,3D* roots (Figure S3d–g, Supporting Information).

We also analyzed whether PIN1 phosphorylation contributed to the effect of ABA on lateral root formation using phospho‐deficient *PIN1‐GFP S1,2,3A* and phospho‐mimic *PIN1‐GFP S1,2,3E* lines.^53^ As seen for gravitropism, the phospho‐deficient *PIN1‐GFP S1,2,3A* line was hypersensitive to ABA in terms of lateral root formation, whereas the phospho‐mimic *PIN1‐GFP S1,2,3E* line showed strong resistance to ABA when compared to that of wild‐type *PIN1‐GFP* plants (Figure 3f). In agreement with ABA effect, inhibition of salt and mannitol on lateral root formation was increased in *PIN1‐GFP S1,2,3A* but repressed in *PIN1‐GFP S1,2,3E* plants (Figure S3h,i, Supporting Information).

These analyses revealed that phosphorylation at Ser 258 (in PIN2) and Ser 252 (in PIN1) was relevant for ABA regulation of root architecture. Notably, the ABA resistance of the phospho‐mimic lines for both root gravitropism and lateral root formation was similar to that of the *1124* ABA‐perception mutant, which showed higher PIN phosphorylation levels (Figure 3a–c). In summary, we identify a specific PIN phosphorylation site that is targeted by PYLs‐dependent ABA signaling and mediates ABA and stress sensitivity of root architecture.

### 2.4 PIN and PID Interact with Catalytic PP2AC Subunits

Next, we explored the mechanism by which ABA modulates the PIN phosphorylation status. The finding that the phospho‐deficient mutants of PIN were hypersensitive to ABA, whereas phospho‐mimic mutants were more resistant to ABA, suggests that ABA targeted the PIN dephosphorylation process. The identified ABA‐regulated phosphorylation site has been proposed to be a target of PID kinase^51^, ^53^ and it has been reported that the PP2A complex acts antagonistically to the PID kinase to influence PIN phosphorylation status.^55^ Notably, ABA has been shown to rapidly downregulate PP2A activity in plants,^18^, ^60^ suggesting that ABA signaling acts on PIN phosphorylation probably through regulating PP2A activity.

Heterotrimeric PP2A holoenzyme complexes comprise PP2AA scaffolding subunits, PP2AB regulatory subunits, and PP2AC catalytic subunits. In *Arabidopsis*, three genes encode PP2AA, 17 encode PP2AB, and five encode PP2AC subunits.^61^ To test the possibility that PP2ACs and PID act as a kinase/phosphatase pair on common substrates, we performed coimmunoprecipitation (co‐IP) assays in vivo using proteins extracted from the cotransformed *Arabidopsis* protoplasts, and found that PID interacted with PP2AC3 or PP2AC4 (Figure S4a, Supporting Information). This interaction was confirmed using a firefly luciferase complementation imaging (LCI) assay through *Agrobacterium*‐mediated transient coexpression in *Nicotiana benthamiana* leaves (Figure S4b,c, Supporting Information). To further determine the genetic interaction between PP2ACs and PID, we crossed *pp2ac3 pp2ac4* double mutant with *PID* gain‐of‐function lines (*35S::PID*). We observed that *pp2ac3 pp2ac4 35S::PID* seedlings showed more severe phenotypes than either parental line (Figure S4d, Supporting Information). Typically, in contrast to *pp2ac3 pp2ac4* mutant with the shorter root,^62^ *pp2ac3 pp2ac4 35S::PID* seedlings arrested growth and even failed to establish root elongation (Figure S4d, Supporting Information). These analyses strongly suggest that PP2AC phosphatases and PID kinase represent antagonistically acting regulators on the common substrates.

To monitor whether PIN could serve as a direct substrate of this kinase/phosphatase pair, we next verified the interaction between the PIN2 hydrophilic loop and all five PP2AC subunits by co‐IP assays (Figure S4e,f, Supporting Information). The PIN1 hydrophilic loop also interacted with the PP2A catalytic subunit as confirmed by yeast two‐hybrid (Y2H) assays (Figure S4g, Supporting Information). Due to the proposed dominant roles of PP2AC3 and PP2AC4 in controlling auxin distribution,^63^ we focused on these subunits for further functional characterizations.

Collectively, these results indicate that the C3 and C4 subunits of PP2A interact with PID kinase to act directly on the common substrates including the PIN proteins.

### 2.5 The PYLs ABA Receptor Directly Regulates PP2A Activity

**Figure 4:**
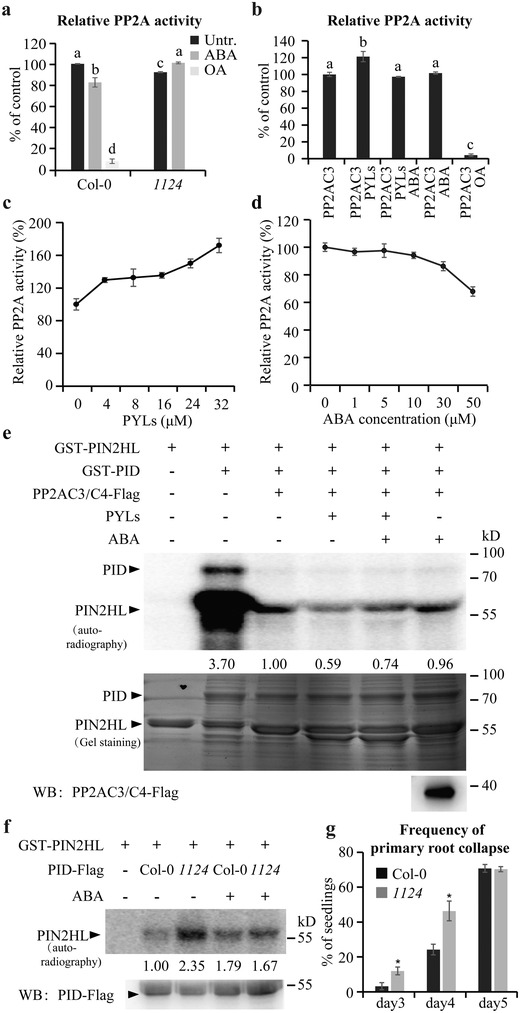
PYLs regulate PP2A activity and thus PID‐mediated PIN phosphorylation. a) An in vivo PP2A activity assay. The total protein used for the assay was extracted from 10‐d‐old seedlings treated or not with 30 × 10^−6^ m ABA for 4 h. The activity of PP2A in untreated wild type was set to 100%. b) An in vitro enzyme activity assay showing the effect of PYLs and/or ABA on the PP2A activity of PP2AC3. The concentrations of PYLs and ABA were 8 × 10^−6^ and 5 × 10^−6^ m, respectively. The phosphatase activity of PP2AC3 alone was set to 100%. PP2A activity was always measured after addition of 1 × 10^−3^ m EDTA to inhibit the activity of PP2C (a,b). OA was used as a phosphatase inhibitor (a,b). Error bars represent ± SE of three replicates. Means with different letters (a,b) are significantly different at *P* < 0.05 (Fisher LSD test). c) PYLs enhancing the phosphatase activity of PP2AC3 in a dosage‐dependent manner in vitro. The concentrations of PYLs protein were 0, 4 × 10^−6^, 8 × 10^−6^, 16 × 10^−6^, 24 × 10^−6^, and 32 × 10^−6^ m, respectively. The phosphatase activity of PP2AC3 alone was set to 100%. d) ABA dosage‐dependent reduction of the PYLs‐increased PP2AC3 activity in vitro. 4 × 10^−6^ m PYLs protein was incubated with different concentrations of ABA before mixing with PP2AC3 for phosphatase activity assay. The phosphatase activity of the sample without ABA treatment was set to 100%. Error bars represent ± SE of three replicates (c,d). e) An in vitro phosphorylation assay reconstituting reversible PIN2HL phosphorylation by ABA, PYLs, and/or PP2ACs. The PP2AC3/C4 expressed in *Arabidopsis* protoplasts, and recombinant PYLs proteins (PYR1, PYL1, PYL2, PYL4), PIN2HL, and PID expressed in *E. coli* were used (b–e). The concentrations of PYLs and ABA used for the phosphorylation assay were 30 × 10^−6^ and 50 × 10^−6^ m, respectively. Arrowheads mark positions of the different proteins. f) PID‐mediated PIN phosphorylation affected by ABA as well as its receptor PYLs. Protoplasts from wild type and *1124* mutant expressing PID‐Flag were treated or not with 10 × 10^−6^ m ABA for 4 h. PID‐Flag was immunoprecipitated and coincubated with purified recombinant GST‐PIN2HL protein from *E. coli* in a phosphorylation reaction. The proteins were finally separated by SDS‐PAGE. Arrowheads mark positions of the different proteins. Numbers under lanes indicate relative band intensities normalized to the loading controls (e,f). + and − indicate incubated with or without substrate, extracts, or ABA treatment, respectively (e,f). g) Quantification of *35S::PID*‐mediated root collapse in 3‐, 4‐, and 5‐d‐old seedlings (*n* ≥ 120 roots). The collapse was significantly promoted by *PYLs* mutation. Error bars represent ± SE of three replicates. (∗) *P* < 0.05 (Student's *t*‐test). Three independent experiments were performed with similar results. Representative images are shown.

Because PP2A provides a possible link between ABA signaling and PIN phosphorylation, we assessed whether PYLs‐dependent ABA signaling could regulate PP2A activity. Total proteins were extracted from the whole seedlings of wild type and the *1124* mutant and analyzed for PP2A activity. Consistent with the PIN phosphorylation, ABA significantly lowered PP2A activity in the wild type, whereas in the *1124* mutant with compromised PYLs receptors, PP2A activity was lower than that of the wild type in the absence of ABA, and ABA treatment elevated PP2A activity (**Figure** **4**a). Besides, the PP2A enzymatic activity was gradually down‐regulated in ABA receptor mutants *pyl1*, *1124*, and *112458* (sextuple mutant lacking *PYR1, PYL1, PYL2, PYL4, PYL5*, and *PYL8*) (Figure S5a, Supporting Information). These results suggest that PYLs‐mediated ABA signaling indeed regulates PP2A activity directly or indirectly.

To substantiate these findings, we performed an in vitro assay to test whether ABA and its receptor PYLs can directly affect the protein phosphatase activity of PP2AC3‐ or PP2AC4‐based PP2A complexes. Four PYLs proteins were purified from *E. coli* and PP2ACs were purified from *Arabidopsis* protoplasts to ensure holoenzyme complex formation. The PP2A activity increased markedly when PYR1, PYL1, PYL2, and PYL4 proteins were together added to the purified PP2AC3 or PP2AC4 complexes in the reaction (Figure 4b; Figure S5b, Supporting Information). In the presence of ABA, PYLs no longer stimulated PP2A activity. ABA alone, without PYLs, had no effect on PP2A activity (Figure 4b; Figure S5b, Supporting Information). We next analyzed the effect of different PYLs/PP2AC ratios on enzymatic activity of PP2A. We again confirmed that activity of PP2AC3 immunoprecipitated from *Arabidopsis* protoplasts could be gradually promoted with increasing amount of heterologously expressed PYLs proteins (Figure 4c; Figure S5c, Supporting Information). This PYLs‐mediated increase in PP2AC3 activity was again attenuated in response to different dose of ABA (Figure 4d). We further measured PP2AC3 phosphatase activity for using the different concentrations of phosphopeptide substrate. A kinetic‐dependent assay indicated that the *K*_m_ of PP2AC3 in the absence or presence of PYLs, or with ABA treatment was 19.9 × 10^−6^, 14.1 × 10^−6^, or 21.0 × 10^−6^ m, respectively (Figure S5d, Supporting Information). These data support our finding that PYLs directly stimulate the activity of PP2ACs, and that ABA interfered with the action of PYLs on PP2ACs. As a control, we examined the effects of ABA receptors on PP2C activity. In contrast to PP2A, the activity of ABI1 was much more greatly inhibited by ABA‐bound PYR1 with increasing amounts of recombinant PYR1 protein (Figure S5e, Supporting Information).^64^ These in vitro assays suggest that the regulation of PYLs and ABA on PP2ACs activity is not as saturable as that of PP2C family members.

To further verify that ABA attenuates the PYLs‐mediated activation of PP2ACs, we reconstituted a reversible PIN phosphorylation system in vitro. We first demonstrated that PID underwent autophosphorylation and mediated the phosphorylation of GST‐PIN2HL (Figure 4e). Both these reactions were substantially counteracted by incubating PP2ACs‐Flag proteins with PID before the kinase assay (Figure 4e). PP2ACs added after PIN2 phosphorylation by PID was effective as well in decreasing the level of phosphorylation (Figure S5f, Supporting Information), suggesting PP2ACs could directly dephosphorylate PID or PIN proteins. As a control, the presence of okadaic acid (OA), an inhibitor of the protein Ser/Thr phosphatases PP1 and PP2A, efficiently blocked PIN dephosphorylation (Figure S5g, Supporting Information), in agreement with previous reports.^65^ Additional incubation of PP2ACs with PYLs further reduced PIN2 phosphorylation levels when compared with the sample without PYLs (Figure 4e), consistent with the promotion of PP2A activity. This PYLs‐mediated decrease in PIN2 phosphorylation was significantly recovered when the protein mix was incubated with ABA (Figure 4e). Importantly, in the absence of PYLs, ABA was incapable of changing the PP2A/PID‐mediated (de)phosphorylation status of PIN2 (Figure 4e). We then added different amounts of PYLs into the reaction mixture and detected that PID‐mediated phosphorylation of PIN2 gradually decreased with increasing amounts of PYLs protein (Figure S5h, Supporting Information). Without PP2AC proteins, PYLs was not able to directly influence PIN2 phosphorylation (Figure S5h, Supporting Information).

To test the effect of ABA and its receptor PYLs on the activity of PP2ACs in plant cells, wild‐type and *1124* protoplasts were transiently transformed with *35S::PID‐Flag* plasmid. The PID‐Flag protein was immunoprecipitated from these protoplasts and coincubated with heterologously expressed GST‐PIN2HL in a phosphorylation reaction. We found that phosphorylation of PIN2 protein was much stronger in the *1124* mutant than that in the wild type. When the protoplasts were treated with 10 × 10^−6^ m ABA for 4 h, PIN2 phosphorylation was increased in wild type but obviously decreased in *1124* (Figure 4f).

These phosphorylation assays are consistent with results from in vivo and in vitro PP2A activity measurements, which showed that regulation of PIN phosphorylation status was dependent on ABA and its receptor PYLs.

To explore the genetic interaction of PYLs and PID that regulating the phosphorylation of PIN proteins, we crossed *1124* into *35S::PID* background and analyzed root collapse caused by auxin depletion from the root meristem.^52^, ^66^ We observed that the *35S::PID*‐mediated collapse was significantly enhanced in *1124* roots when compared to that of wild type (Figure 4g). These genetic analyses are consistent with the biochemical studies showing that PYLs regulate PIN phosphorylation.

### 2.6 The PYLs ABA Receptor Interacts with Catalytic PP2AC Subunits

**Figure 5:**
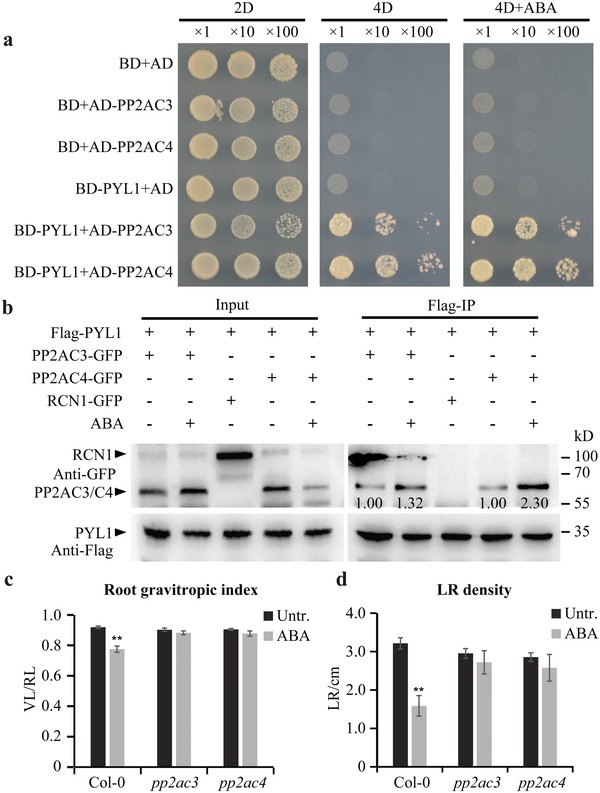
PYLs interact with PP2ACs. a) A yeast two‐hybrid assay showing PYL1 interacting with PP2AC3/C4 and ABA enhancing this interaction. 2D synthetic dropout medium lacks Trp/Leu and 4D selective medium lacks Trp/Leu/His/Ade. 50 × 10^−6^ m ABA was applied. b) A co‐IP assay showing PYL1 interacting with PP2AC3/C4. As a negative control, PYL1 did not coimmunoprecipitate with RCN1. Treatment with 10 × 10^−6^ m ABA for 4 h led to enhanced interaction between PYL1 and PP2AC3/C4. Numbers under lanes indicate relative band intensities normalized to the loading controls. + and − indicate incubated with or without extracts or ABA treatment. c,d) Increased tolerance of *pp2ac3* and *pp2ac4* roots in response to ABA. The root gravitropic index (c) and lateral root density (d) were analyzed (*n* ≥ 10 roots). Data are means ± SE, (∗∗) *P* < 0.01 (Student's *t*‐test). Three independent experiments were performed with similar results. Representative images are shown.

The ABA‐dependent PYLs action on PP2A activity in vitro suggested a direct interaction between PYLs and PP2AC subunits. Through Y2H assays, we found that PYL1 indeed directly interacted with PP2AC3 or PP2AC4 subunits, and these interactions were promoted by ABA treatment (**Figure** **5**a). The co‐IP assays showed that PYR1, PYL1, PYL2, and PYL4 coimmunoprecipitated with PP2AC3 or PP2AC4 in vivo (Figure 5b; Figure S6a,b, Supporting Information). Moreover, the interaction between PYL1 and PP2AC3 or PP2AC4 could be enhanced by addition of ABA (Figure 5b). The LCI assay validated that coexpression of PYL1 with PP2AC4 resulted in strong complementation of LUC activity that was markedly increased by the addition of ABA (Figure S6c,d, Supporting Information). In addition, an in vitro pull‐down assay verified that the GST‐PP2AC3 or GST‐PP2AC4 proteins, but not GST alone, was able to pull down the PYL1‐His protein (Figure S6e, Supporting Information). These observations collectively reveal that PYR1/PYL1/PYL2/PYL4 ABA receptors physically interact with PP2AC3 or PP2AC4 and these interactions are sensitive to ABA. This probably explains the antagonistic functions of PYLs on PP2A activity in the presence and absence of ABA. Given their functional redundancy,^25^ it is likely that other PYLs members interact with PP2ACs.

These data reveal a mechanism by which PYLs interact with PP2ACs and regulate their activity, consistent with observations that mutants of different PP2A subunits have altered sensitivity to ABA in seed germination, stomata closure, cotyledon expansion, and root growth.^17^, ^18^, ^20^ This mechanism could also explain how ABA regulates root architecture in response to salt or osmotic stress. To examine this further, we analyzed the effect of ABA on root growth of *pp2ac3* and *pp2ac4* mutants. We first confirmed that *pp2ac3 pp2ac4* double mutants showed decreased PP2A activity that was no longer sensitive to ABA (Figure S6f, Supporting Information). We next performed an in vitro phosphorylation assay to examine phosphorylation status of PIN2 in *pp2ac3 pp2ac4* double mutant background. Consistent with the PP2A activity measurements (Figure S6f, Supporting Information), the total proteins extracted from the *pp2ac3 pp2ac4* double mutant had a greater ability to phosphorylate GST‐PIN2HL than those extracted from the wild type (Figure S6g, Supporting Information). Although ABA treatment markedly enhanced phosphorylation of GST‐PIN2HL in the wild type; when proteins extracted from the *pp2ac3 pp2ac4* mutant were used, we did not observe this dramatic ABA effect (Figure S6g, Supporting Information). Accordingly, in comparison with the wild type, impairment of *PP2AC3* and *PP2AC4* genes that expressed in the roots (Figure S6h, Supporting Information) led to reduced sensitivity to the ABA‐mediated inhibition in root gravitropic response (Figure 5c; Figure S6i, Supporting Information) and lateral root formation (Figure 5d; Figure S6j, Supporting Information). Moreover, both *pp2ac3* and *pp2ac4* mutants showed enhanced salt‐tolerance compared to wild type in terms of root gravitropic bending (Figure S6k, Supporting Information) and seedling survival rate (Figure S6l, Supporting Information). The ABA‐ and stress‐hyposensitive phenotypes of *pp2ac3* and *pp2ac4* overlapped with those of mutants defective in *PYL*s, all of which displayed reduced abilities to dephosphorylate PIN proteins. By contrast, *pp2ac1* and *pp2ac5*, together with knock‐down mutant *pp2ac2* (Figure S6m, Supporting Information), showed almost similar sensitivities to ABA as wild type in terms of root gravitropic responses (Figure S6n, Supporting Information) and lateral root formation (Figure S6o,p, Supporting Information).^18^

Together, these observations indicate a mechanism in which PYLs interact with catalytic subunits C3 and C4 of PP2A and regulate their phosphatase activity. By such a mechanism, roots growing in natural conditions can flexibly adapt to increased salt or adverse osmotic conditions in the soil.

## 3 Discussion

**Figure 6:**
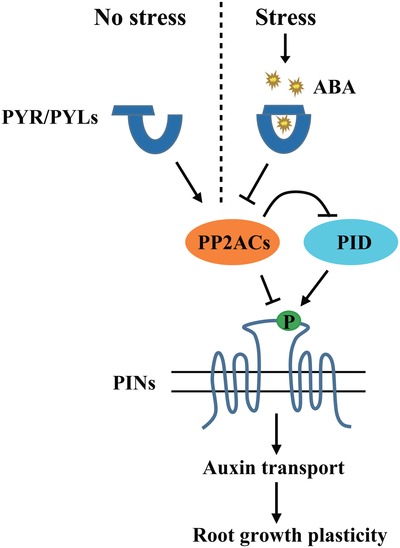
Proposed model illustrating stress‐ and ABA‐regulated adaptive root architecture. In the absence of stress, PYLs directly interact with PP2A catalytic subunits and promote PP2A activity. This counteracts PID kinase‐mediated PIN phosphorylation. Thus, PIN‐dependent auxin transport functions normally. In the presence of stress, rapidly accumulated ABA binds to the PYLs receptors and PP2A activity is inhibited. The phosphorylation of PIN proteins is increased and in turn auxin transport activity is modulated. Ultimately, root growth plasticity is regulated in response to environmental cues by this adaptive mechanism.

In this study, we revealed that plants reorient their growth direction or reprogram organogenesis to withstand saline and osmotic stresses by an adaptive regulatory mechanism mediated by ABA signaling. We found that ABA bound to PYLs under stress and then PP2A activity was inhibited, which increased PID‐mediated PIN phosphorylation and thereby modulated PIN‐directed auxin transport, ultimately resulting in adaptive root development. By contrast, in unstressed plants, PYLs promoted PP2A activity and maintained a relatively lower level of PIN phosphorylation and normal PIN‐dependent auxin transport, which supported normal root development (**Figure** **6**). In the current model of ABA signaling, PYLs together with canonical PP2C coreceptors are considered core regulators of the ABA signaling module from ABA perception to downstream gene expression.^6^, ^7^ Here, we found that root gravitropism and lateral root formation are regulated via a PYLs‐PP2A complex when plants are exposed to saline and osmotic stresses. Our results support that PP2C phosphatase ABI1 is not involved in this novel ABA pathway. A total of nine protein phosphatases are classified to clade A PP2Cs in *Arabidopsis* 7 and we cannot exclude the signals through other PP2Cs might also participate in regulating root adaptive development. In addition to the classical ABA signaling pathway, our study revealed a novel adaptive mechanism involving the PYLs‐PP2A complex by which root growth and development was adjusted to withstand stresses in the soil.

Five genes in the *Arabidopsis* genome encode catalytic subunits of PP2A, which can be divided into two subfamilies based on their sequence conservation: subfamily I, including PP2AC1, PP2AC2, and PP2AC5; and subfamily II, including PP2AC3 and PP2AC4.^67^ Our Y2H analysis revealed that all five catalytic subunits of PP2A interacted with PYL1 (Figure 5a; Figure S7, Supporting Information), yet only *pp2ac3* and *pp2ac4* mutants, as *pyls* higher‐order mutants displayed reduced responses to ABA and stresses. Consistent with previous data,^18^, ^62^, ^63^, ^68^ our results suggest functional diversification between these two subfamilies, indicating that only subfamily II members were involved in this PYLs‐PP2A regulatory mechanism, but three other PP2AC subunits of subfamily I might play distinct biological functions in the ABA pathway, which is still undefined. Together with previous reports that PP2AC3 and PP2AC4 redundantly modulate embryonic patterning and root development through regulating auxin gradients,^63^ our experimental results link intercellular auxin fluxes with the ABA signaling pathway. Our data also highlight the importance of the catalytic subunits of the PP2A holoenzyme complex. In addition, all the data support that altered PIN phosphorylation status could lead to the different response of *pp2ac3* and *pp2ac4* mutants to ABA from the wild type, although we cannot exclude the effects of other components in the classical ABA signal transduction pathway. Nonetheless, the phosphor‐mutants of PINs clearly show that the effect of ABA signaling on PIN phosphorylation is the major mechanism for remodeling the root growth by ABA.

PIN‐mediated auxin transport is regulated by PP2A phosphatase and AGCVIII kinase family, including D6 PROTEIN KINASE (D6PK) and PID/WAG kinases, through the reversible phosphorylation.^55^, ^57^, ^58^ Besides, PIN proteins have also been reported to be phosphorylated by MITOGEN‐ACTIVATED PROTEIN KINASEs (MPKs), which inhibit the polar targeting and thus the function of PINs.^69^, ^70^ However, it is still unclear which phosphorylation on which sites within PIN sequences contributes to which extent to the PIN activity and/or PIN polar localization. Although the rootward (still basipetal) auxin transport is reduced in the *d6pk* stem,^57^ hyperphosphorylation of PIN proteins upon ABA treatment through repressing dephosphorylation, leading to decreased basipetal auxin transport as well (Figure 2a), probably interfered with PIN activity, which could be tightly controlled by the balance of PIN phosphorylation status. In our study, we focus on the regulation of PIN phosphorylation from the PP2A phosphatase (through dephosphorylation) side. We have tested PID‐mediated PIN phosphorylation and also known PID‐targeted phosphosites, which together verified that PID indeed plays a vital role in the ABA‐PYLs‐PP2A pathway. However, it is likely that more protein kinases of the different types are involved in controlling PIN localization and activity, and can be counteracted by the PP2A phosphatases, which typically have broader substrate specificity. Despite the details remain not entirely clear, our study show that ABA‐regulated PIN phosphorylation is a critical part of the mechanism, by which ABA regulates plant adaptive development.

Our LC‐MS/MS‐based analysis, in vitro phosphorylation and Phos‐tag assays showed that PIN phosphorylation was increased by ABA treatment in the wild type but decreased in the *1124* mutant (Figure 3a–c). Similarly, ABA treatment reduced in vivo PP2A activity in the wild type but promoted activity in the *1124* mutant (Figure 4a). It should be noted that proteins extracted from whole plants were used in these experiments. However, in the absence or presence of heterologously expressed PYLs, no opposite effects of ABA treatment were observed from in vitro PP2A activity measurement (Figure 4b; Figure S5b, Supporting Information) and PIN phosphorylation assays (Figure 4e). Therefore, it is possible that these contrasting effects of ABA in the wild type and *1124* mutant might be caused by feedback regulation in the ABA signaling pathway or mediated by other unknown factors that could regulate PP2A activity in planta. Together, these results suggest that ABA positively regulates PIN phosphorylation through PYLs receptors.

ABA perception through PYLs is evolutionarily conserved. The phosphorylated PIN proteins that over‐accumulated in the *pyls* higher‐order mutants caused the agravitropic root response and aberrant lateral root development, similar phenotypes as previously observed in *pp2aa* multiple mutants or *PID* overexpression lines.^51^, ^55^, ^66^ The *pp2ac3* and *pp2ac4* mutants and PIN phosphomimic lines exhibited relatively hyposensitive phenotypes to ABA, in line with the phenotypes of the *pyl*s quadruple mutant in root developmental context. The PINs are direct substrates of PP2AC3 and PP2AC4, and PP2AC3 and PP2AC4 interact with PID, thus PP2AC3 and PP2AC4 might play an antagonistic role with PID in regulating ABA‐mediated PIN relocation, auxin gradient, and subsequent adaptive root development. Unlike the dramatic basal‐to‐apical PIN polarity shift in *pp2aa* loss‐ and *PID* gain‐of‐function mutants,^51^, ^52^, ^55^ the effects of ABA signaling on PIN distribution were relatively moderate. Accordingly, this might not be the only mechanism and other signals might also be involved. In this study, we unraveled a novel stress‐ and ABA‐mediated regulatory mechanism that contributes to adaptive root development. Potential molecular components of the PYLs‐PP2A pathway and the mechanism by which PYLs select PP2A or PP2C remain to be elucidated. Collectively, our study provides insight into the adaptive responses of roots to external stimuli. These findings lay the foundation for the targeted engineering of root architecture to improve plant tolerance to stresses in the environment.

## Conflict of Interest

The authors declare no conflict of interest.

## Supporting information

Supporting Information
