# Supplementary material for: Root Growth Adaptation is Mediated by PYLs ABA Receptor‐PP2A Protein Phosphatase Complex
Source: Adv Sci (Weinh). 2019 Dec 11;7(3):1901455. doi: 10.1002/advs.201901455 (PMC7001640; doi:10.1002/advs.201901455)
Supplement: Supplementary file 1 — Supporting Information [file ADVS-7-1901455-s001.pdf]

Copyright WILEY-VCH Verlag GmbH & Co. KGaA, 69469 Weinheim, Germany, 2019.

ADVANCED SCIENCE
Open Access

Supporting Information

for *Adv. Sci.,* DOI: 10.1002/advs.201901455

Root Growth Adaptation is Mediated by PYLs ABA Receptor-PP2A Protein Phosphatase Complex

*Yang Li, Yaping Wang, Shutang Tan, Zhen Li, Zhi Yuan, Matouš Glanc, David Domjan, Kai Wang, Wei Xuan, Yan Guo, Zhizhong Gong, Jiří Friml, and Jing Zhang**

Supporting Information

Root Growth Adaptation is Mediated by PYLs ABA Receptor-PP2A Protein Phosphatase Complex

*Yang Li, Yaping Wang, Shutang Tan, Zhen Li, Zhi Yuan, Matouš Glanc, David Domjan, Kai Wang, Wei Xuan, Yan Guo, Zhizhong Gong, Jiří Friml, and Jing Zhang**

Figure S1

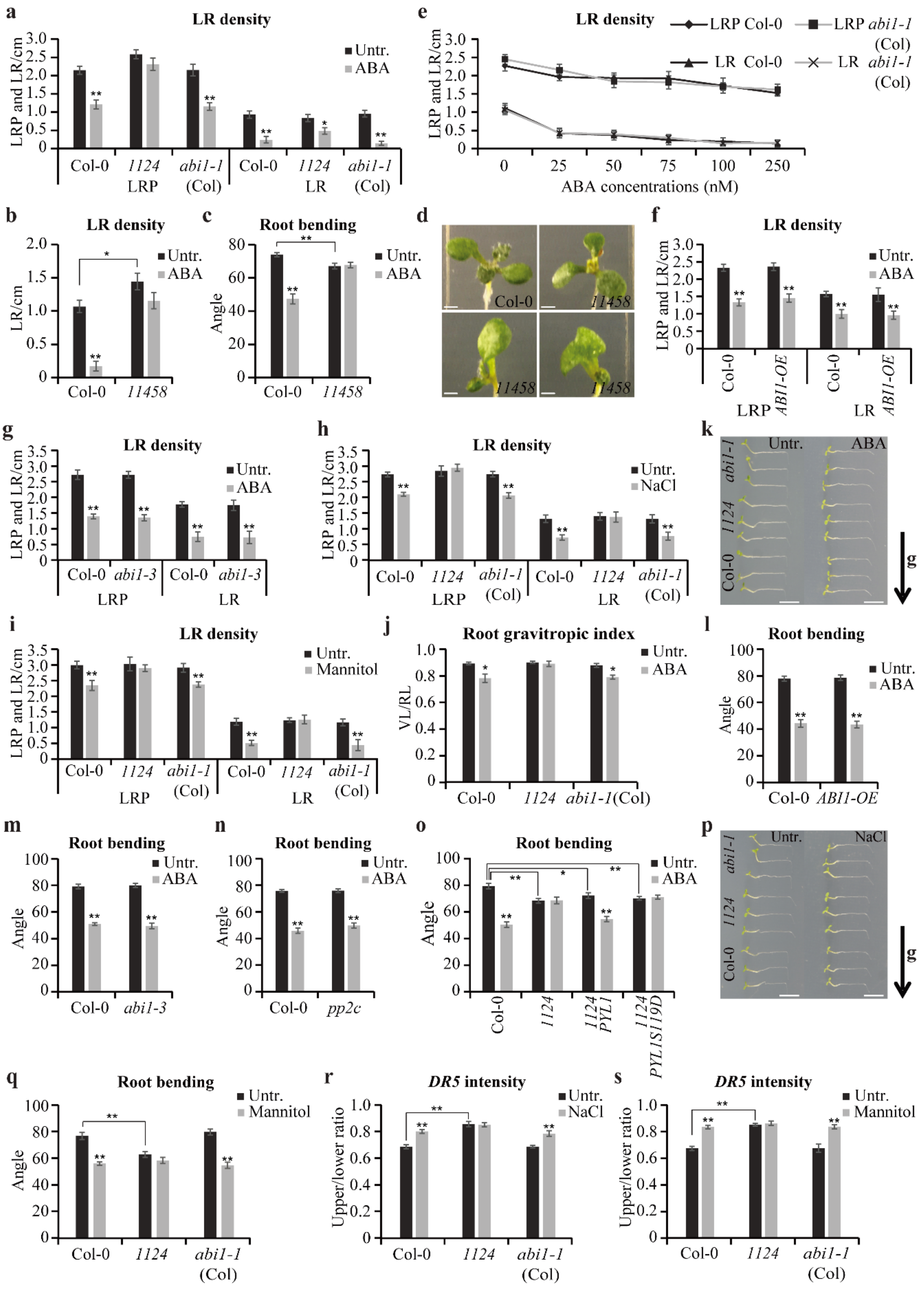

Figure S1. ABA and stresses affect root architecture. a) The effect of ABA on lateral root organogenesis. Density of lateral root primordia and emerged lateral root was quantified in 7-

d-old seedlings germinated on MS medium with or without 0.3 × 10^{-6} M ABA (*n* = 12 roots). b-d) Morphological defects of the higher-order knockout mutant *11458*, which shows increased lateral root formation (b), attenuated gravitropic root curvature (c), and aberrant seedling development (3%; *n* = 403 plants), as shown by one, three, and fused cotyledons (d). Besides, ABA-mediated suppression of lateral root formation (b) and root gravity (c) was not observed in *11458* mutant (*n* = 13 roots). Scale bar, 0.1 cm. e) The dosage effect of ABA on lateral root formation in the *abi1-1* (Col-0) mutant, which remains similar to that of the wild-type seedlings (*n* = 12 roots). f,g) ABA response in the *ABI1*-overexpressing (*ABI1-OE*) plants (f) and *abi1-3* mutant (g), which exhibits similar ABA sensitivity to the wild type regarding lateral root organogenesis (*n* ≥ 11 roots). h,i) The effect of NaCl (h) and mannitol (i) on lateral root organogenesis. Lateral root density was quantified in 7-d-old seedlings germinated on MS medium with or without 20 × 10^{-3} M NaCl and 25 × 10^{-3} M mannitol (*n* ≥ 11 roots). j) Quantification of root gravitropic index (vertical length [VL]/root length [RL]). Five-day-old seedlings were exposed to 30 × 10^{-6} M ABA for another 5 d (*n* = 12 roots). k-q) Root gravitropic bending under ABA, NaCl or mannitol treatments. Five-day-old seedlings were gravistimulated in the presence or absence of 30 × 10^{-6} M ABA (k-o), 50 × 10^{-3} M NaCl (p), or 250 × 10^{-3} M mannitol (q) for 24 h (*n* ≥ 10 roots). Scale bar, 0.5 cm. r,s) The effect of NaCl (r) and mannitol (s) on auxin translocation. Five-day-old seedlings transgenically expressing *DR5rev::GFP* were transferred to MS medium supplemented or not with 50 × 10^{-3} M NaCl (r) and 250 × 10^{-3} M mannitol (s) for 16 h and then gravistimulated for 4 h in darkness (*n* ≥ 12 roots). The ratio of mean fluorescence intensity of the upper to that of lower side of root was quantified. Error bars represent ± SE (a-c,e-j,l-o,q-s). (∗) *P* < 0.05, (∗∗) *P* < 0.01 (Student's *t*-test). Three independent experiments were performed with similar results. Representative images are shown.

Figure S2

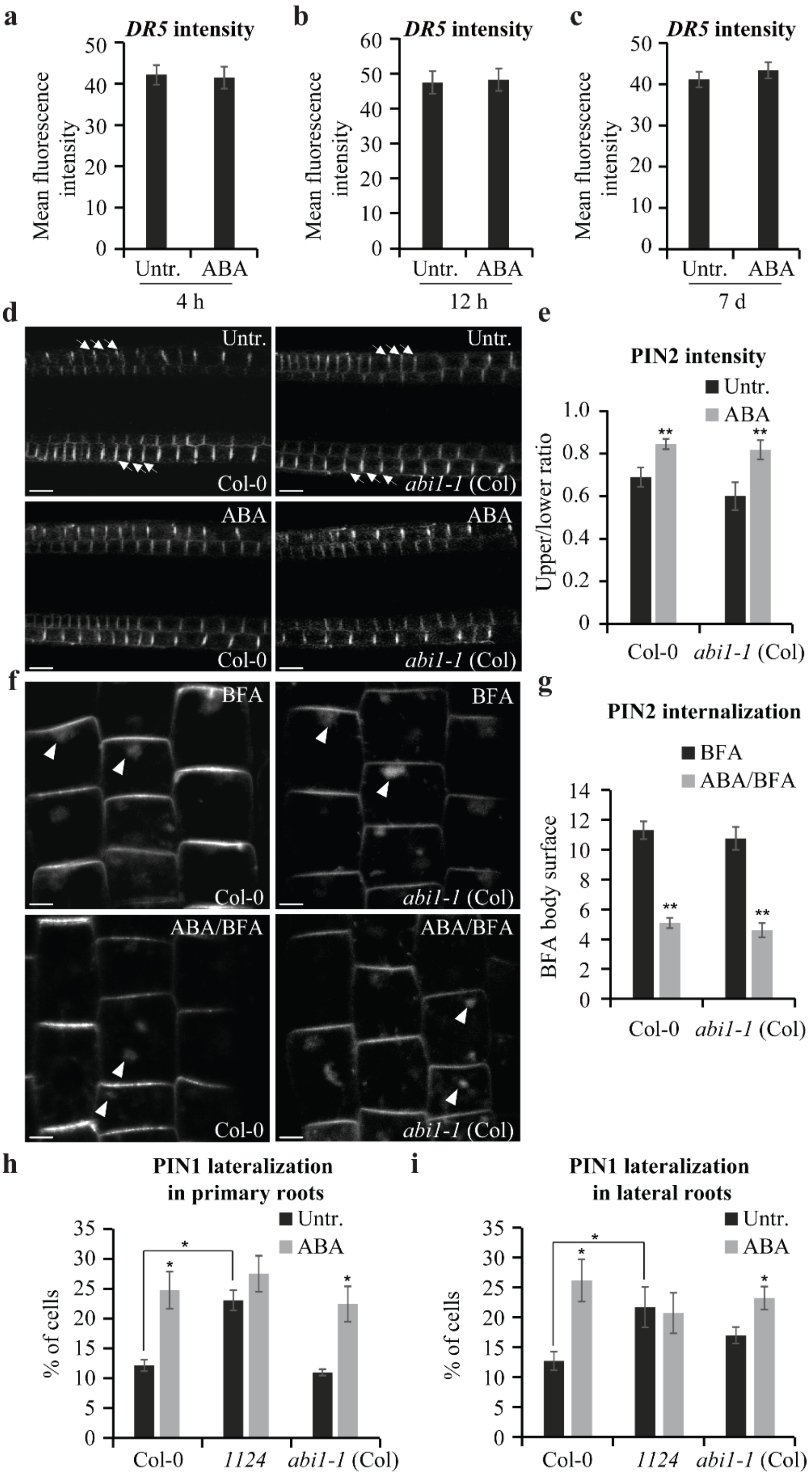
a
DR5 intensity
Mean fluorescence intensity
Untr. ABA
4 h
b
DR5 intensity
12 h
c
DR5 intensity
7 d
d
Untr.
Col-0
abi1-1 (Col)
ABA
e
PIN2 intensity
Untr.
ABA
Upper/lower ratio
Col-0 abi1-1 (Col)
f
BFA
ABA/BFA
g
PIN2 internalization
BFA
ABA/BFA
BFA body surface
h
PIN1 lateralization in primary roots
% of cells
Col-0 1124 abi1-1 (Col)
i
PIN1 lateralization in lateral roots

Figure S2. ABA affects PIN distribution and subcellular dynamics. a-c) Quantification of *DR5rev::GFP* intensity in the root tip. Five-day-old seedlings were treated or not with 30 × 10^{-6} M ABA for 4 h (a) or 12 h (b) and 7-d-old seedlings were germinated on MS medium supplemented or not with 0.3 × 10^{-6} M ABA (c) ($n \geq 10$ roots). d,e) The effect of ABA on gravistimulation-mediated PIN2 gradient. Relative PIN2-GFP intensity in the epidermis at the upper versus lower side of the root was quantified (e; $n = 10$ roots). Arrows mark the PIN2 gradient. Scale bar, 20 µm. f,g) Effect of ABA on BFA-induced PIN2-GFP internalization. Mean surface area (pixels^2) of BFA bodies per cell in root epidermis was quantified (g; $n \geq$ 112 cells). Arrowheads mark PIN2 proteins internalized into BFA compartments. Scale bar, 5 µm. h,i) Effect of ABA on PIN1-GFP polar targeting. Seven-day-old seedlings germinated on MS or 0.3 × 10^{-6} M ABA medium. The proportion of cells with rearranged PIN1 in primary roots (h; $n \geq 212$ cells) and emerged lateral roots (i; $n \geq 260$ cells) was quantified. Error bars represent ± SE (a-c,e,g-i). (∗) $P < 0.05$, (∗∗) $P < 0.01$ (Student's *t*-test). Three independent experiments were performed with similar results. Representative images are shown.

Figure S3

a

Position	Score	Expect	Peptide
257~273	96	1.50E-09	ASSFNQTDFYAMFNASK
329~361	40	0.00021	SMSGELYNNNSVPSYPPPNP MFTGSTSGASGVK
382~401	34	0.0006	EMNMFVWSSSASPVSEANAK

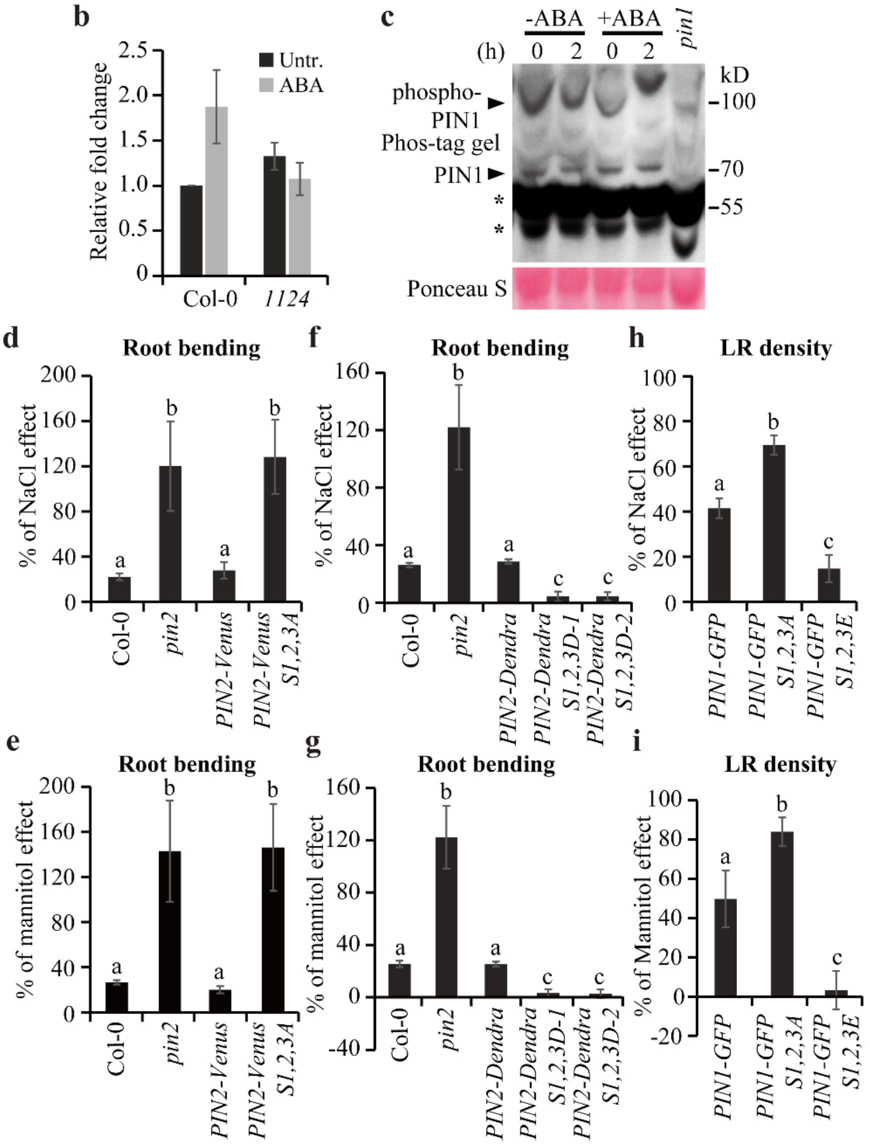

Figure S3. A phosphorylation-based sequence modifies sensitivity to salt and mannitol stress. a) Sequences of recovered peptides from the GST-PIN2HL as derived from LC-MS/MS data. These phosphopeptides all had Mascot score higher than 30 (P <0.05). b) The statistical analysis of the abundance changes of the phosphopeptide 257-273. The fold change values

were averages of three biological replicates. Error bars represent ± SE. c) An in vivo phosphorylation profile after Phos-tag mobility shift assay. Protein extracts from 7-d-old seedlings treated with 30 × 10^{-6} M ABA or ethanol (solvent control) for 2 h were separated in a Phos-tag gel. Strongly shifted upper bands should be phosphorylated PIN1, and the weak lower bands should be non-phosphorylated PIN1. Both phosphorylated and unphosphorylated PIN1 proteins (arrowheads indicated) were detected with anti-PIN1 antibody. *pin1* mutant were used as a negative control. The strong lower nonspecific bands are indicated by asterisks. Considering that all bands shifted abnormally for phos-tag gels, the protein markers do not tell the real mobility for proteins. + and - indicate incubated with or without treatment. d-g) Quantification of root gravitropic bending in phospho-deficient and phospho-mimic lines under salt and mannitol treatments. The root growth angle was measured in 5-d-old seedlings gravistimulated with or without 50 × 10^{-3} M NaCl or 250 × 10^{-3} M mannitol for 24 h ($n \geq 11$ roots). Two independent transgenic lines *PIN2-Dendra S1,2,3D-1* and *PIN2-Dendra S1,2,3D-2* were used (f,g). h,i) Quantification of lateral root density in phospho-deficient and phospho-mimic lines under salt and mannitol treatments. Seven-day-old transgenic lines germinated on MS medium supplemented with or without 20 × 10^{-3} M NaCl or 25 × 10^{-3} M mannitol were used for lateral root density quantification ($n \geq 11$ roots). Error bars represent ± SE. Means with different letters are significantly different at $P < 0.05$ (Fisher LSD test) (d-i). Three independent experiments were performed with similar results.

Figure S4

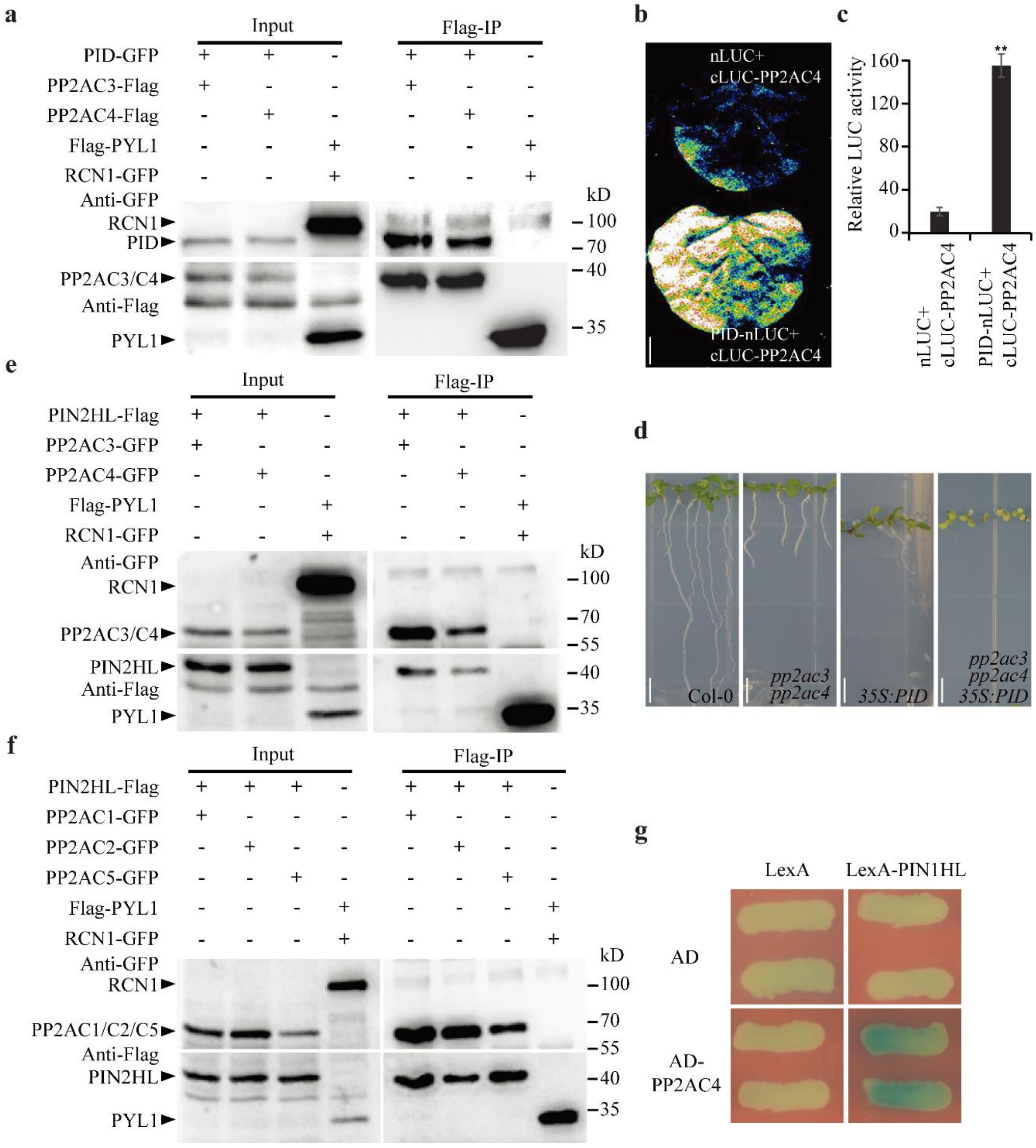

Figure S4. PP2ACs physically or genetically interact with PID and PIN. a) A co-IP assay showing that PID interacts with PP2AC3/C4. As a negative control, PYL1-Flag did not coimmunoprecipitate with RCN1-GFP. The PP2AC3/C4-Flag and PID-GFP plasmids were co-transformed into *Arabidopsis* protoplasts. Total isolated proteins were immunoprecipitated with anti-Flag agarose and detected by immunoblotting with anti-GFP antibody. b,c) A LCI assay showing that PID interacts with PP2AC4 in plant cells. Relative fluorescence was

quantified by ImageJ (c). Scale bar, 1 cm. Error bars represent ± SE. (∗∗) $P < 0.01$ (Student's *t*-test). d) Strongly enhanced phenotypes of *pp2ac3 pp2ac4 35S::PID* seedlings as compared to either parental line of the same age. Scale bar, 0.5 cm. e,f) Co-IP assays showing that PIN2HL interacts with PP2AC3/C4 (e) and also with PP2AC1/C2/C5 (f). As a negative control, PYL1-Flag did not coimmunoprecipitate with RCN1-GFP. The PP2ACs-GFP and PIN2HL-Flag plasmids were co-transformed into *Arabidopsis* protoplasts. Coimmunoprecipitation and immunoblotting analysis were performed as in (a). + and - indicate incubated with or without extracts, respectively (a,e,f). g) A yeast two-hybrid assay showing that PIN1HL interacts with PP2AC4. AD: B42 activation domain; BD: LexA DNA-binding domain. Three independent experiments were performed with similar results. Representative images are shown.

Figure S5

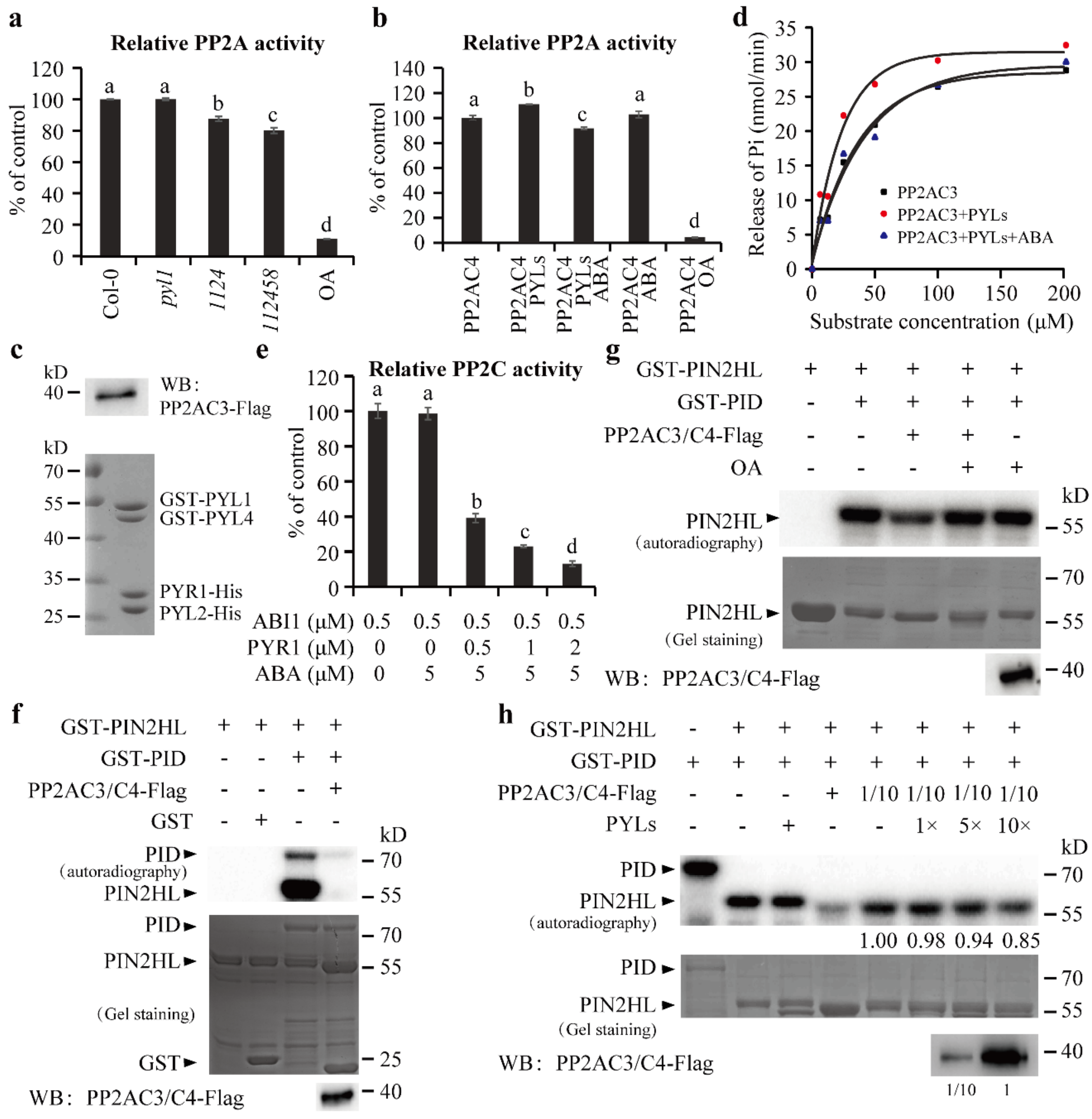

Figure S5. PYLs regulate PP2A activity and thus PID-mediated PIN phosphorylation. a) An in vivo PP2A activity assay. The total protein used for the assay was extracted from 10-d-old seedlings. The phosphatase activity of wild type was set to 100%. b) An in vitro enzyme activity assay of PP2AC4. The concentrations of PYLs and ABA were 8×10^{-6} and 5×10^{-6} M, respectively. The phosphatase activity of PP2AC4 alone was set to 100%. PP2A activity was always measured after addition of 1×10^{-3} M EDTA to inhibit the activities of PP2C (a,b). OA was used as a phosphatase inhibitor (a,b). c) Protein purity of PYLs isolated from *E.coli*

and PP2AC3 immunoprecipitated from *Arabidopsis* protoplasts. These purified proteins were used in different assays. d) Kinetic-dependent curve of PP2AC3 phosphatase activity with or without PYLs and ABA in vitro. PP2AC3 phosphatase activity was measured using different concentrations of substrate phosphopeptide (6.25×10^{-6}, 12.5×10^{-6}, 25×10^{-6}, 50×10^{-6}, 100×10^{-6} and 200×10^{-6} M). The concentration of PYLs was 4×10^{-6} M. The calculated K_m and V_{max} of PP2AC3 alone, PP2AC3 and PYLs without or with ABA treatment was 19.9×10^{-6}, 14.1×10^{-6} or 21.0×10^{-6} M, and 26.9, 31.5 or 27.1 nmol/min, respectively. The data were curve-fitted using the Origin2018 software. e) ABA-bound PYR1 inhibiting the phosphatase activity of ABI1 in vitro. The different molecular ratios between recombinant ABI1 and PYR1 proteins used for the phosphatase activity assay were 1:0, 1:1, 1:2, and 1:4, respectively. The concentration of applied ABA was 5×10^{-6} M. 5×10^{-6} M OA was applied to the reaction to inhibit the activity of PPP family Ser/Thr-specific phosphoprotein phosphatases, such as PP1 and PP2A. The phosphatase activity of untreated ABI1 was set to 100%. f) An in vitro phosphorylation assay showing that PP2ACs added after PIN phosphorylation by PID was effective as well in decreasing the level of phosphorylation. g) An in vitro phosphorylation assay showing that PP2ACs reduced PID-mediated phosphorylation of PIN2HL and the dephosphorylating ability of PP2ACs was inhibited by OA treatment. h) An in vitro phosphorylation assay: PID-mediated PIN phosphorylation was not influenced by addition of PYLs, but strongly reduced by sufficient PP2ACs protein. When the amount of PP2ACs was reduced by 90%, PIN phosphorylation was again recovered. However, gradually increasing amount of PYLs could ultimately lead to decreased PIN phosphorylation. Numbers under lanes indicate relative band intensities normalized to the loading controls. The concentrations of PYLs used for the phosphorylation assay were 0.8×10^{-6}, 4×10^{-6} and 8×10^{-6} M, respectively. + and - indicate incubated with or without substrate, extracts, or OA treatment (f-h). Arrowheads mark positions of the different proteins (f-h). Error bars (a,b,e) represent ± SE of three replicates. Means with different letters are

significantly different at $P < 0.05$ (Fisher LSD test). Three independent experiments were performed with similar results. Representative images are shown.

Figure S6

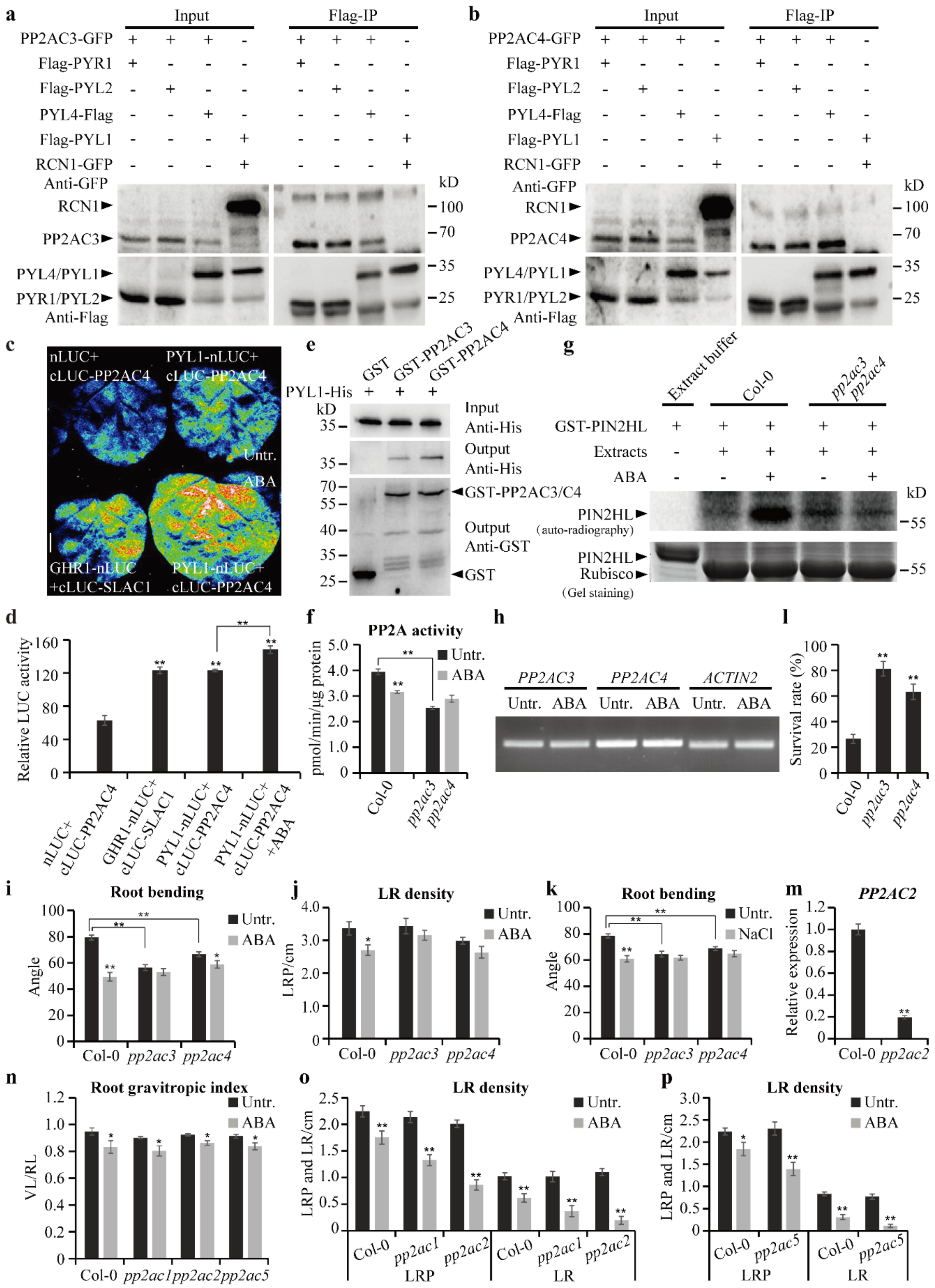

Figure S6. PYLs interact with PP2ACs, and *pp2ac* mutants have diverse sensitivity to ABA.

a,b) Co-IP assays showing that PP2AC3 (a) or PP2AC4 (b) interacts with PYR1, PYL2, and

PYL4, respectively. c,d) A LCI assay showing that PYL1 interacts with PP2AC4 and this interaction is enhanced by ABA. As a positive control, GHR1 interacts with SLAC1. Scale bar, 1 cm. For ABA treatment, the tobacco leaves were sprayed with 100 × 10^{-6} M ABA. Relative fluorescence was quantified by ImageJ (d). e) In vitro pull-down assay showing the interaction of PYL1 and PP2AC3 or PP2AC4. PYL1-His, GST-PP2AC3, GST-PP2AC4, and GST proteins were expressed in *E. coli*. Purified proteins were used for the pull-down assay. His-tagged PYL1 protein pulled down with GST, GST-PP2AC3, or GST-PP2AC4 was detected by anti-His antibody. f) An in vivo PP2A activity assay in the *pp2ac3 pp2ac4* double mutant. g) An in vitro phosphorylation assay of PIN2HL in the *pp2ac3 pp2ac4* double mutant. Equal amounts of total protein extracts from 5-d-old seedlings treated or not with 30 × 10^{-6} M ABA for 4 h were co-incubated with heterologously expressed GST-PIN2HL, and then used for an in vitro phosphorylation assay. + and - indicate incubated with or without substrate, extracts, or ABA treatment, respectively (a,b,e,g). h) mRNA detection of *PP2AC3* and *PP2AC4* in wild-type roots by reverse transcription polymerase chain reaction (RT-PCR). *ACTIN2* was used as a reference control gene. RNA was isolated from roots of 5-d-old wild-type seedlings treated or not with 30 × 10^{-6} M ABA for 4 h. i,j) Increased tolerance of *pp2ac3* and *pp2ac4* mutants to the ABA-mediated inhibition in root gravitropic bending (i) and lateral root primordia development (j) ($n \geq 10$ roots). k) NaCl-insensitive phenotypes of *pp2ac3* and *pp2ac4* mutants in terms of root gravitropic bending ($n = 13$ roots). l) Tolerance of *pp2ac3* and *pp2ac4* mutants under salt stress. Five-day-old seedlings grown on MS medium were transferred onto MS medium supplemented with 175 × 10^{-3} M NaCl for 4 d. Survival rates of *pp2ac3* and *pp2ac4* were evaluated after salt treatment ($n = 180$ seedlings). m) *PP2AC2* transcription in wild type and *pp2ac2* mutant detected by quantitative real-time PCR (qRT-PCR). The expression of *PP2AC2* in the wild type was set to 1. *ACTIN2* is the internal control. RNA was isolated from 5-d-old seedlings. n-p) ABA response in *pp2ac1*, *pp2ac2*, and *pp2ac5* mutants. Sensitivity of *pp2ac1*, *pp2ac2*, and *pp2ac5* to ABA is almost similar to

that of wild type in root gravitropism (n) and lateral root organogenesis (o, p**)** (*n* ≥ 10). Error bars (d,f,i-p) represent ± SE. (∗) $P < 0.05$, (∗∗) $P < 0.01$ (Student's *t*-test). Three independent experiments were performed with similar results. Representative images are shown.

Figure S7

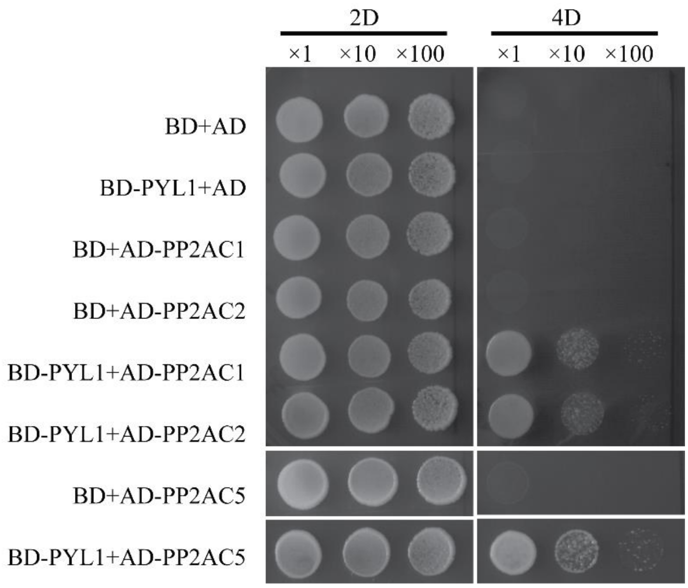

Figure S7. PYL1 interacts with PP2ACs. Yeast two-hybrid assay showing the interaction of PYL1 with PP2AC1/C2/C5. Three independent experiments were performed with similar results. Representative images are shown.

Table S1. Primers used for cloning.

Primer name	Restriction enzymes	Primer sequence (5'-3')
pCAMBIA-PP2AC3-F	SmaI	ATGGGCGCGAATTCG
pCAMBIA-PP2AC3-R	SpeI	AAGGAAATAGTCAGGTGTCCTT
pCAMBIA-PP2AC4-F	SmaI	ATGGGCGCGAATTCTATTC
pCAMBIA-PP2AC4-R	SpeI	CAGGAAATAGTCTGGAGTCCTT
pCAMBIA-PIN2HL-F	SmaI	ATGCAGTTCCCGGAGACGGCTG
pCAMBIA-PIN2HL-R	SpeI	CGGCGGCATCTGCTGTTTC
pCAMBIA-PID-F	SmaI	ATGTTACGAGAATCAGACGGTGA
pCAMBIA-PID-R	SpeI	AAAGTAATCGAACGCCGCTG
pCAMBIA-RCN1-F	SmaI	ATGGCTATGGTAGATGAACCGTT
pCAMBIA-RCN1-R	SpeI	GGATTGTGCTGCTGTGGAA
35S-PYL1-F	XbaI	ATGGCGAATTCAGAGTCCTC
35S-PYL1-R	XmaI	TTACCTAACCTGAGAAGAGTTGT
35S-PYR1-F	XbaI	ATGCCTTCGGAGTTAACACC
35S-PYR1-R	XmaI	TCACCTGAGAACCA
35S-PYL2-F	XbaI	ATGAGCTCATCCCCGG
35S-PYL2-R	XmaI	TTATTCATCATCATGCATAGGTGCAG
pCAMBIA-PYL4-F	SmaI	ATGCTTGCCGTTCACCG
pCAMBIA-PYL4-R	SpeI	CAGAGACATCTTCTTCTTGCTCTCAG
pGBKT7-PYL1-F	SmaI	AATGGCGAATTCAGAGTCCTC
pGBKT7-PYL1-R	BamHI	TTACCTAACCTGAGAAGAGTTGTTGT
pGADT7-PP2AC1-F	EcoRI	ATGCCGTTAAACGGAGATCT
pGADT7-PP2AC1-R	BamHI	TCACAAAAAATAATCAGGGGTCTTG
pGADT7-PP2AC2-F	EcoRI	ATGCCGTCGAACGGA
pGADT7-PP2AC2-R	BamHI	TCACAAAAAATAATCAGGGGTCTTC
pGADT7-PP2AC5-F	SmaI	AATGCCGCCGGC
pGADT7-PP2AC5-R	BamHI	TTACAAAAAATAATCTGGAGTCTTGC
pGADT7-PP2AC3-F	SmaI	AATGGGCGCGAATTCG
pGADT7-PP2AC3-R	BamHI	TCAAAGGAAATAGTCAGGTGTC
pGADT7-PP2AC4-F	SmaI	AATGGGCGCGAATTCTATTC
pGADT7-PP2AC4-R	BamHI	TCACAGGAAATAGTCTGGAGTC
pCAMBIA-PP2AC1-F	SmaI	ATGCCGTTAAACGGAGATCT
pCAMBIA-PP2AC1-R	SpeI	CAAAAAATAATCAGGGGTCTTGCG

pCAMBIA-PP2AC2-F	SmaI	ATGCCGTCGAACGGA
pCAMBIA-PP2AC2-R	SpeI	CAAAAAATAATCAGGGGTCTTCCG
pCAMBIA-PP2AC5-F	SmaI	ATGCCGCCGGC
pCAMBIA-PP2AC5-R	SpeI	CAAAAAATAATCTGGAGTCTTGCG
cLUC-PP2AC4-F	KpnI	ATGGGCGCGAATTCTATTC
cLUC-PP2AC4-R	BamHI	TCACAGGAAATAGTCTGGAGTC
PID-nLUC-F	KpnI	ATGTTACGAGAATCAGACGGTGA
PID-nLUC-R	SalI	AAAGTAATCGAACGCCGCTG
PYL1-nLUC-F	KpnI	ATGGCGAATTCAGAGTCCTC
PYL1-nLUC-R	SalI	CCTAACCTGAGAAGAGTTGTTGT
B42AD-PP2AC4-F	MfeI	ATGGGCGCGAATTCTATTC
B42AD-PP2AC4-R	XhoI	TCACAGGAAATAGTCTGGAGTC
LexA-PIN1HL-F	EcoRI	ATGTACCGTGGAGCTAAGCTTTTG
LexA-PIN1HL-R	XhoI	ACTGGAGTAAGAGTTGGGATTAC

Table S2. Primers used for genotyping.

Primer name	Primer sequence (5'-3')
PP2AC1-LP	ACAGGTTTTCTGTTTGCATGG
PP2AC1-RP	TCGATGCCTTATAACAACGAAG
PP2AC2-LP	TGGGGTATTTAGGCACAAATG
PP2AC2-RP	GTTTCTGGCTGATCAGCAAAG
PP2AC3-LP	GCTTGAAAGAACAGCATTTCG
PP2AC3-RP	GTGGATTATCACCATCCATCG
PP2AC4-LP	TAATTGGTATCAGGGCACTGC
PP2AC4-RP	TGTTTCCTGATCTGTTTTCCG
PP2AC5-LP	ATTGAATCCAAACACAAACCG
PP2AC5-RP	TCAAGTCCAAAACAGCAGAAAC

Table S3. Primers used for qRT-PCR and RT-PCR.

Primer name	Primer sequence (5'-3')
PP2AC2-RT-F	TGATCGATGTGGATGGGGAA
PP2AC2-RT-R	CACCACATTCTTATCCTGACACC
PP2AC3-RT-F	TCCATCGAGACCCTTGACAA

PP2AC3-RT-R	TGTTCCGAAATATCCTGACCAA
PP2AC4-RT-F	ACAGCCTTGGTTGAGTCAGA
PP2AC4-RT-R	GAGAGATGCCCCAACCACAT
ACTIN2-F (At3g18780)	GCACCCTGTTCTTCTTACCG
ACTIN2-R (At3g18780)	AACCCTCGTAGATTGGCACA

Detailed Methods

Materials

The following mutants and transgenic plants were described previously: *abi1-1* (Col-0);[71-73] *ABI1-OE*; [27,31] *abi1-3* (SALK_076309);[32] *abi1 abi2 hab1*;[35] *1124*, *11458* and *112458*;[24,25] *1124-PYL1* and *1124-PYL1S119D*;[36] *pp2ac1* (SALK_102599);[63] *pp2ac3* (SALK_069250) and *pp2ac4* (SALK_035009);[62] *pp2ac5* (SALK_139822);[20,63] *PIN1::PIN1-GFP*;[49] *PIN2::PIN2-GFP*;[74] *PIN2::PIN2-Dendra*;[75] *DR5rev::GFP*;[76] *35S::PID*;[66] *pin1* (*pin1-613*);[77] *pin2* (*eir1-1*);[40] *PIN2::PIN2-Venus* and *PIN2::PIN2-Venus S123A*;[51] and *PIN1::PIN1-GFP S123A* and *PIN1::PIN1-GFP S123E*.[53] The insertion mutant line *pp2ac2* (SALK_139685) was obtained from Nottingham Arabidopsis Stock Center (NASC). The downregulation of the *PP2AC2* transcript was shown by qRT-PCR.

The *PIN2::PIN2-Dendra S1,2,3D* transgenic plants were engineered as follows: a 1033-bp N-terminal fragment of the *PIN2* CDS in which three Ser residues, Ser 237 (TCA 709-711), Ser 258 (TCT 772-774), and Ser 310 (TCA 928-930), were converted to Asp (GAT) was commercially synthesized by Eurofins (Germany). A C-terminal fragment containing the Dendra tag was amplified from the original *PIN2::PIN2-Dendra* construct.[75] Overlap-PCR was then used to synthesize full-length *PIN2* cDNA by fusing N- and C-terminal fragments, which was then introduced into a Gateway-compatible entry vector pDONR221. The resulting clone together with a P4-P1R entry clone containing the *PIN2* promoter[75] was assembled simultaneously into the Gateway destination vector pB7m24GW,3. The obtained *PIN2::PIN2-Dendra S1,2,3D* construct was transformed into *pin2* plants by floral dip.[78]

The oligonucleotides used for PCR in this study are listed in Table S1-S3.

Experimental conditions and drug applications

Arabidopsis thaliana plants were grown on soil or regular MS medium containing 1% sucrose (w/v) and 1% agar (w/v) at pH 5.8 under a 16/8-h light/dark photoperiod at 20-22°C. *Nicotiana benthamiana* plants were grown in soil under the same conditions.

Exogenous drugs were applied by incubating seedlings in solid or liquid MS medium supplemented with ABA (30×10^{-3} M stock in ethanol; Sigma) (0.3×10^{-6} / 30×10^{-6} M, unless otherwise noted) or BFA (25×10^{-3} M stock in DMSO; Sigma) (25×10^{-6} M). For all other biochemical studies, the concentrations of ABA applied were 1×10^{-6}, 5×10^{-6}, 10×10^{-6}, 30×10^{-6}, 50×10^{-6} or 100×10^{-6} M as specified. Control treatments always contained an equivalent amount of solvent.

To test ABA inhibition on BFA-induced internalization, 5-d-old seedlings were treated for 1 h with 25×10^{-6} M BFA or pretreated for 3 h with 30×10^{-6} M ABA, followed by a 1 h co-treatment of ABA/BFA. For transient protoplast assays, 4-week-old seedlings horizontally grown on MS medium were used. For PP2A activity assays, 10-d-old seedlings grown on MS medium were treated for 4 h with 30×10^{-6} M ABA or the equivalent amount of ethanol. For other experiments, unless otherwise noted, 5-d-old seedlings were always grown vertically on MS supplemented with 0.3×10^{-6} M ABA or the equivalent amount of ethanol.

Root gravitropism evaluations

Five-day-old seedlings grown vertically on MS medium were transferred to solid medium supplemented with 30×10^{-6} M ABA or the equivalent amount of ethanol as control. This concentration is commonly used for root-bending assay.[79-81] For root gravitropic index assays, the started positions of the root tips were marked and the plates were photographed after 5 d of vertical growth on ABA or control medium. Downward growth was recorded from the marked point. The vertical length and root length were measured with ImageJ (https://imagej.nih.gov/ij/download.html), and a root vertical growth index (VGI = vertical

length / root length) was calculated as described previously.[82] For root gravitropic bending assays, the plates were immediately turned 90° compared with the original vertical position. After 24 h of the gravistimulation in darkness, the root growth angle was measured using ImageJ.[34] For the *DR5* translocation assays, seedlings were gravistimulated at 90° for 4 h in darkness after 16 h of vertical growth on ABA or control medium. For PIN2 translocation assays, seedlings were gravistimulated at 90° for 4 h in darkness immediately after transferring to ABA or control medium. Confocal imaging was performed immediately and ImageJ was used to measure fluorescence intensity.

Lateral root density evaluations

7-day-old seedlings germinated on the MS medium supplemented or not with 0.3×10^{-6} M ABA. Lateral root initiation and developmental progression analyses were performed as described previously.[49,83]

Auxin transport assays

Basipetal auxin transport was measured in 6-d-old plants as described.[84] Briefly, the seedlings were firstly treated for 12 h in liquid MS medium supplemented with 30×10^{-6} M ABA or the equivalent amount of ethanol. After transferring these seedlings onto solid ABA or control medium, agar droplets containing [^{3}H]IAA (25 Ci/mmol or 1 mCi/mL American Radiolabeled Chemicals) were applied to the apical 0.5 mm of the root tip. Following 5-6 h of incubation in darkness at room temperature, around 5 mm segments at a distance of 2 mm from the [^{3}H]IAA application sites were harvested. Radioactivity was measured with a scintillation counter (HIDEX 300SL).

In situ expression and localization analyses

Whole-mount immunolocalization in *Arabidopsis* roots was performed as described.[85] The anti-PIN2 antibody (1:1000; provided by C. Luschnig) and secondary antibody anti-TRITC (1:600; Sigma) were used. For in vivo GFP inspections, seedlings were mounted in liquid MS medium containing the relevant drugs or an equivalent amount of solvent without fixation for live-cell imaging. All fluorescence signals were evaluated on Carl Zeiss 710 or Leica SP5 confocal scanning microscopes. The confocal imaging was always performed at the same position of roots with the same microscope settings for each independent experiment. All the cells in the field of vision were analyzed for quantification of PIN distribution and BFA-mediated PIN internalization. Images were finally assembled in Adobe Photoshop 7.0 and Adobe Illustrator CS4.

Y2H assays

The full-length coding sequences of *PYL1* and *PP2AC*s were fused into pGBKT7 (binding domain, BD) and pGADT7 (activation domain, AD) vectors, respectively. These plasmids were co-transformed into yeast strain AH109 according to the standard yeast PEG transformation method. Transformed yeast cells were separately sprayed onto 2D synthetic dropout medium lacking Trp/Leu and 4D selective medium lacking Trp/Leu/His/Ade, and incubated for 4-5 d at 30°C. For ABA treatment, 4D selective medium was supplemented with 50×10^{-6} M ABA. LexA-based Y2H assays were performed as described previously.[86]

Co-IP assays

Full-length *PYR1*, *PYL1*, and *PYL2* were fused into the 35S::Flag vector with an N-terminal Flag tag, and *PYL4*, *PP2ACs*, *PID*, and *PIN2HL* were fused into the pCAMBIA1300 vector

with a C-terminal GFP or Flag tag. The primers used to clone the constructs are listed in Table S1. *Arabidopsis* wild-type protoplasts transformed with an equivalent amount of the relevant constructs were incubated in 1 mL of W5 buffer (154×10^{-3} M NaCl, 125×10^{-3} M $CaCl_2$, 5×10^{-3} M KCl, and 2×10^{-3} M MES [pH 5.7]) for 14-16 h and subsequently lysed in 1 mL of protein extraction buffer (50×10^{-3} M Tris-Cl [pH 7.4], 150×10^{-3} M NaCl, 2×10^{-3} M EDTA, 0.5% Triton X-100, protease inhibitor cocktail, and 1×10^{-3} M PMSF). After 15 min of centrifugation (12,000 *g*) at 4°C, 80 μL of supernatant was reserved as input and the remaining part was immunoprecipitated with anti-Flag agarose (Sigma) for 3 h at 4°C. The beads were then washed 5-6 times with washing buffer (50×10^{-3} M Tris-Cl [pH 7.4], 150×10^{-3} M NaCl, 2×10^{-3} M EDTA, 0.1% Triton X-100, protease inhibitor cocktail, and 1×10^{-3} M PMSF). The immunoprecipitates were then separated on a 10% SDS-PAGE gel and the targeted proteins were detected with anti-GFP antibody (Sigma).

In vitro pull-down assays

The pull-down assay was performed as previously described with some modifications.[31] Briefly, the full-length coding sequence of PP2AC3, PP2AC4 or PYL1 was cloned into the pGEX-4T-1 vector or the pET28a vector. GST, GST-PP2AC3, GST-PP2AC4 proteins were purified on Glutathione Sepharose 4B beads (GE) and PYL1-His protein was purified on Ni Sepharose (GE). 10 μg of GST or GST-PP2AC3, GST-PP2AC4 proteins was incubated with 10 μL Glutathione Sepharose 4B beads mixed with 1×PBS buffer containing 0.1% Triton X-100 at 4°C for 1 h. The buffer was then removed after centrifugation. 1 μg of PYL1-His protein mixed with the fresh binding buffer was incubated at 4°C and rotated for 2 h. The beads were subsequently washed four times with 1×PBS buffer containing 0.1% Triton X-100. The pulled-down proteins were eluted in 5 × SDS loading buffer at 100°C for 5 min, and then separated on 10% SDS-PAGE gels, eventually detected by immunoblotting.

LCI assays

The full-length coding sequence of *PP2AC4* was amplified and fused to the C-terminal of the pCAMBIA-cLUC vector, and the full-length coding sequence of *PYL1* and *PID* were amplified and fused to the N-terminal of the pCAMBIA-nLUC vector, respectively. The LCI assay was performed as previously described.[87] All fusions were transformed into *Agrobacterium* strain GV3101. Positive clones were cultured in YEB liquid medium overnight at 28°C. The relevant bacteria were mixed, centrifuged (10000 *g*; 3 min), resuspended in activity buffer (10×10^{-3} M $MgCl_2$, 10×10^{-3} M MES, and 150×10^{-6} M acetosyringone, pH 5.7) and incubated for 2-5 h at room temperature. Subsequently, young leaves of *Nicotiana benthamiana* plants were infiltrated with the bacteria and plants were grown for another 2 d before harvesting. For ABA treatment, the infiltrated leaves were sprayed with 100×10^{-6} M ABA and incubated for 1 h at room temperature. The abaxial sides of infiltrated leaves were sprayed with 1×10^{-3} M LUC substrate (D-luciferin) before the LUC signal was captured with a CCD camera following 15 min of a dark incubation period at room temperature.

In vitro phosphorylation assays and mass spectrometry analyses

The *His-PIN1HL* and *GST-PIN2HL* C-terminal fusions were constructed by fusing the hydrophilic loops of PIN1 and PIN2 into the pET30a and pGEX-4T-1 vectors, respectively. *Escherichia coli* BL21-Codon Plus strain (DE3) cells were used for the heterologous expression of His-PIN1HL and GST-PIN2HL. Total proteins were extracted from 5-d-old seedlings with 1× kinase buffer (25×10^{-3} M Tris-HCl, pH 7.5, 1×10^{-3} M DTT, and 5×10^{-3} M $MgCl_2$), 1× protease inhibitor cocktail (Roche), and 1×10^{-3} M PMSF. For in vitro phosphorylation, approximately 2 μg of purified protein of His-PIN1HL and 25 μg of plant

extract were added to the kinase reaction mix, containing 1× kinase buffer and 1× ATP solution (100 × 10^{-6} M ATP and 1 μCi [γ-^{32}P] ATP), in a total volume of 25 μL. The reactions were incubated at 30°C for 30 min, terminated by the addition of 6.25 μL of 5× protein loading buffer (310 × 10^{-3} M Tris-HCl [pH 6.8]; 10% SDS; 50% glycerol; 750 × 10^{-3} M β-mercaptoethanol; 0.125% bromophenol blue), and then incubated at 100°C for 5 min. Reactions were subsequently separated through 12% acrylamide gels, and stained with Coomassie Brilliant Blue. The radiolabeled signals were captured using a Typhoon scanner (GE Amersham) with a storage phosphor-screen after exposure for 48 h. For mass spectrometry (LC-MS/MS), GST-PIN2HL was used as substrate, and the phosphorylation reaction was performed as for the in vitro phosphorylation assay with plant extracts, but radiolabeled ATP was excluded. After Coomassie staining, the target bands were cut out and subjected to trypsin digestion. Peptides were then separated by nano-liquid chromatography and subjected to tandem mass spectrometry.

The other phosphorylation assays without plant extracts were performed as previously described.[55] Since PP2ACs were purified from *Arabidopsis* protoplasts, the amount of these proteins was always evaluated by the western blot. For phosphorylation reactions, anti-Flag beads associated with PP2ACs protein extracted from protoplasts were mixed with purified recombinant protein PYLs incubated or not with 50 × 10^{-6} M ABA for 15 min at room temperature. Unless otherwise noted, PIN2HL pre-incubated with PID in the kinase buffer was then added into the reaction. After incubation at 30°C for 30 min, the supernatant was subsequently analyzed by SDS-PAGE.

Phos-tag assays

To examine the phosphorylation status of PIN1 in vivo, a mobility shift based Phos-tag SDS-PAGE method was used. The homozygous *pin1* mutant seedlings selected based on the

deficient cotyledon phenotype were always included as the control. Seven-day-old seedlings were treated with 30×10^{-6} M ABA or ethanol (solvent control) respectively for 2 h. 100 mg tissue collected into a 2 mL Eppendorf tube was thoroughly ground with an ironic grinding ball in liquid nitrogen and was then homogenized in 100 µL protein extraction buffer (50×10^{-3} M Tris-HCl pH7.5, 150×10^{-3} M NaCl, 0.5% [v/v] Triton X-100, protease inhibitor cocktail [cOmplete, Sigma, #11836170001], and phosphatase inhibitor cocktail [PhosSTOP, Sigma, #4906837001]). Samples were incubated on ice for 20 min, and afterwards were spun down at 4 °C at 14, 000 rpm for 10 min. The supernatant (100 µL) was transferred to a new 1.5 mL Eppendorf tube and was then added with 25 µL 5× SDS loading buffer (0.312 M Tris-HCl pH6.8, 10% [w/v] SDS, 250×10^{-3} M DTT, 50% [v/v] glycerol, 0.01% [w/v] Bromophenol Blue). For dephosphorylation assay, 20 µL protein extracts were added with 20 U CIAP (Calf Intestinal Alkaline Phosphatase, Thermo Fisher Scientific, #18009019), and incubated at 37°C for 30 min. 15 µL of each protein sample was separated by 50×10^{-6} M Zn^{2+} Phos-tag (Phos-tag acrylamide AAL-107, Wako Pure Chemical Industries, #304-93521) SDS-PAGE (prepared according to the manufacturer's manual). After transfer, the PVDF membrane was blocked by 5% (w/v) skim milk in 1× TBST (50×10^{-3} M Tris-HCl, 150×10^{-3} M NaCl, 0.1% [v/v] Tween-20, pH 7.6) and incubated with primary (Rabbit anti-PIN1, 1:1000) and secondary (Donkey anti-rabbit IgG antibody conjugated with horseradish peroxidase [HRP], 1:15000, GE Healthcare, #NA934) antibodies sequentially.[38] Finally, the signal was detected by the SuperSignal™ West Femto Maximum Sensitivity Substrate (ThermoFisher Scientific, #34095), and imaged by a Peqlab Fusion SL Advance device.

In vivo and in vitro PP2A activity measurements

The Serine/Threonine Phosphatase Assay System (Promega) was used to determine the PP2A activity according to the manufacturer's instructions. For the in vivo assays, PP2A activity

was measured in total protein extracts from 10-d-old seedlings. 1 × 10^{-3} M EDTA was always added to the reaction to inhibit the activity of PP2C. For in vitro assays, *PYL*s were cloned into the pET28a or pGEX-4T-1 vector. Recombinant proteins of PYLs-His or GST-PYLs were expressed in *E. coli* BL21 strain and extracted as described for in vitro phosphorylation assays. *Arabidopsis* wild-type protoplasts were transiently transformed with *35S::PP2AC3-Flag* and *35S::PP2AC4-Flag*, and proteins were extracted as described for co-IP assays. After washing anti-Flag beads for 5-6 times, elution buffer (containing 100-500 μg mL^{-1} Flag peptide in TBS buffer) was mixed with the anti-Flag beads, which was incubated at 4°C and rotated for 2 h. The elution buffer was then collected by centrifugation and used for PP2A activity measurement. The samples were prepared in phosphate-free water to a final volume of 35 μL, containing 10 μL of PP2ACs protein and the indicated amounts of PYLs incubated or not with ABA. After incubation at room temperature for 15 min, the samples were mixed with specific PP2A reaction buffer (250 × 10^{-3} M imidazole [pH7.2], 1 × 10^{-3} M EGTA, 0.1 % β-mercaptoethanol and 0.5 mg/mL BSA) and synthetic phosphopeptide substrate RRA[pT]VA. 1 × 10^{-3} M EDTA was always added to the reaction to inhibit the activity of PP2C. The mixtures were incubated at 37°C for 30 min before adding 50 μL of molybdate dye/additive mix to terminate the reactions. After incubation at room temperature for 20 min, color was developed and absorbance at 600 nm was measured with a BioTek Microplate spectrophotometer (Power wave XS2). The phosphate released by samples was determined based on the standard curve prepared using 0, 100, 200, 500, 1000, and 2000 pmol phosphate solutions.

In vitro PP2C activity measurements

PP2C activity was measured using a Serine/Threonine Phosphatase Assay System (Promega) as described previously.[36] The buffer used for PP2C activity measurement contained 250 ×

10^{-3} M imidazole [pH7.2], 1 × 10^{-3} M EGTA, 25 × 10^{-3} M $MgCl_2$, 0.1 % β- mercaptoethanol, and 0.5 mg/mL BSA. 5 × 10^{-6} M okadaic acid was always applied to the reaction to inhibit the activity of PPP family Ser/Thr-specific phosphoprotein phosphatases, such as PP1 and PP2A.

References

[71] J. Leung, M. Bouvier-Durand, P. C. Morris, D. Guerrier, F. Chefdor, J. Giraudat, *Science* **1994**, *264*, 1448.
[72] X. Luo, Z. Chen, J. Gao, Z. Gong, *Plant J.* **2014**, *79*, 44.
[73] K. Meyer, M. P. Leube, E. Grill, *Science* **1994**, *264*, 1452.
[74] J. Xu, B, Scheres, *Plant Cell* **2005**, *17*, 525.
[75] Y. Salanenka, I. Verstraeten, C. Löfke, K. Tabata, S. Naramoto, M. Glanc, J Friml, *Proc. Natl. Acad. Sci. USA* **2018**, *115*, 3716.
[76] J. Friml, A. Vieten, M. Sauer, D. Weijers, H. Schwarz, T. Hamann, R. Offringa, G. Jürgens, *Nature* **2003**, *426*, 147.
[77] T. Bennett, T. Sieberer, B. Willett, J. Booker, C. Luschnig, O. Leyser, *Curr. Biol.* **2006**, *16*, 553.
[78] S. J. Clough, A. F. Bent, *Plant J.* **1998**, *16*, 735.
[79] J. He, Y. Duan, D. Hua, G. Fan, L. Wang, Y. Liu, Z. Chen, L. Han, L. -J. Qu, Z. Gong, *Plant Cell* **2012**, *24*, 1815.
[80] L. Yang, J. Zhang, J. He, Y. Qin, D. Hua, Y. Duan, Z. Chen, Z. Gong, *PLoS Genet*. **2014**, *10*, e1004791.
[81] H. Yin, X. Zhang, J. Liu, Y. Wang, J. He, T. Yang, X. Hong, Q. Yang, Z. Gong, *Plant Cell* **2009**, *21*, 386.
[82] A. Grabov, M. K. Ashley, S. Rigas, P. Hatzopoulos, L. Dolan, F. Vicente-Agullo, *New Phytol.* **2005**, *165*, 641.
[83] A. Bielach, K. Podlešáková, P. Marhavý, J. Duclercq, C. Cuesta, B. Müller, W. Grunewald, P. Tarkowski, E. Benková, *Plant Cell* **2012**, *24*, 3967.
[84] D. R. Lewis, G. K. Muday, *Nat. Protoc.* **2009**, *4*, 437.
[85] M. Sauer, T. Paciorek, E. Benková, J. Friml, *Nat. Protoc.* **2006**, *1*, 98.
[86] S. Zhang, C. Li, Y. Zhou, X. Wang, H. Li, Z. Feng, H. Chen, G. Qin, D. Jin, W. Terzaghi, H. Gu, L.-J. Qu, D. Kang, X. W. Deng, J. Li, *Plant Cell* **2018**, *30*, 835.
[87] H. Chen, Y. Zou, Y. Shang, H. Lin, Y. Wang, R. Cai, X. Tang, J. -M. Zhou, *Plant Physiol.* **2008**, *146*, 368.
